# Engineered Radiative Cooling Systems for Thermal-Regulating and Energy-Saving Applications

**DOI:** 10.1007/s40820-025-01859-1

**Published:** 2025-08-05

**Authors:** Leqi Lei, Ting Wu, Shuo Shi, Yifan Si, Chuanwei Zhi, Kaisong Huang, Jieqiong Yang, Xinshuo Liang, Shanshan Zhu, Jinping Qu, Jinlian Hu

**Affiliations:** 1 Department of Biomedical Engineering, City University of Hong Kong, Hong Kong, 999077 Hong Kong SAR People’s Republic of China; 2 School of Chemistry and Chemical Engineering, Huazhong University of Science and Technology Wuhan, Hubei, 430074 People’s Republic of China; 3 School of Fashion and Textiles, The Hong Kong Polytechnic University, Hong Kong, Hong Kong SAR People’s Republic of China; 4 Guangdong-Hong Kong-Macao Joint Laboratory of Human-Machine Intelligence-Synergy Systems, Shenzhen Institutes of Advanced Technology, Chinese Academy of Sciences, Shenzhen, 518055 People’s Republic of China

**Keywords:** Radiative cooling systems, Engineered materials, Thermal-regulating, Energy-saving, Smart applications

## Abstract

**Highlights:**

- This review thoroughly encapsulates the contemporary advancements in radiative cooling systems, from materials to applications.
- Comprehensive discussion of the fundamental concepts of radiative cooling systems, engineered materials, thermal-regulating textiles and energy-saving devices.
- The review critically evaluates the obstacles confronting radiative cooling systems, offering insightful and forward-looking solutions to shape the future trajectory of the discipline.

**Abstract:**

Radiative cooling systems (RCSs) possess the distinctive capability to dissipate heat energy via solar and thermal radiation, making them suitable for thermal regulation and energy conservation applications, essential for mitigating the energy crisis. A comprehensive review connecting the advancements in engineered radiative cooling systems (ERCSs), encompassing material and structural design as well as thermal and energy-related applications, is currently absent. Herein, this review begins with a concise summary of the essential concepts of ERCSs, followed by an introduction to engineered materials and structures, containing nature-inspired designs, chromatic materials, meta-structural configurations, and multilayered constructions. It subsequently encapsulates the primary applications, including thermal-regulating textiles and energy-saving devices. Next, it highlights the challenges of ERCSs, including maximized thermoregulatory effects, environmental adaptability, scalability and sustainability, and interdisciplinary integration. It seeks to offer direction for forthcoming fundamental research and industrial advancement of radiative cooling systems in real-world applications.

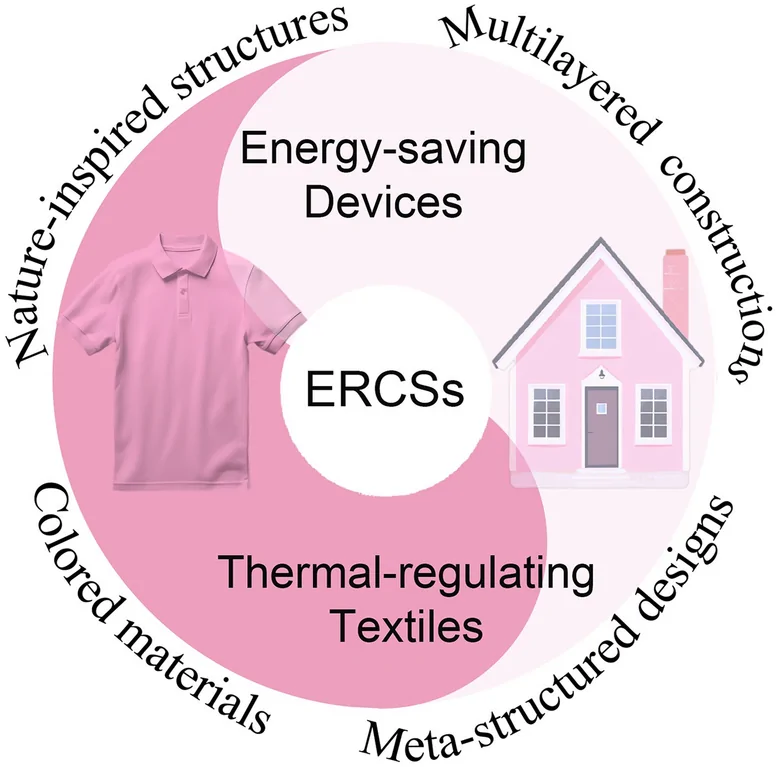

## Introduction

Over the years, rapid population growth and intensive electricity consumption make the earth warmer yearly in recent decades, especially in hot summer. However, commonly employed cooling approaches based on vapor compression, such as air conditioning systems and the fans, consume a substantial electricity produced from electricity power [1–5]. This escalating energy consumption results in an expanding output of greenhouse gas emissions, which in turn contributes to a constant elevation in global temperature. Moreover, radiative cooling system (RCS) is crucial for providing thermal comfort in living areas and motivating to create more eco-friendly and energy-efficient effects for different applications, such as buildings (e.g., roof, windows), vehicles, industrial equipment, and personal thermal management [6–12]. Studies suggest that it is preferable to demonstrate a particular technology than to modify the window of heat dissipation due to variations in thermal cooling effect. Consequently, the implementation of a cost-effective and innovative RCS has the potential to significantly reduce global energy usage [6, 13–18].

**Fig. 1:**
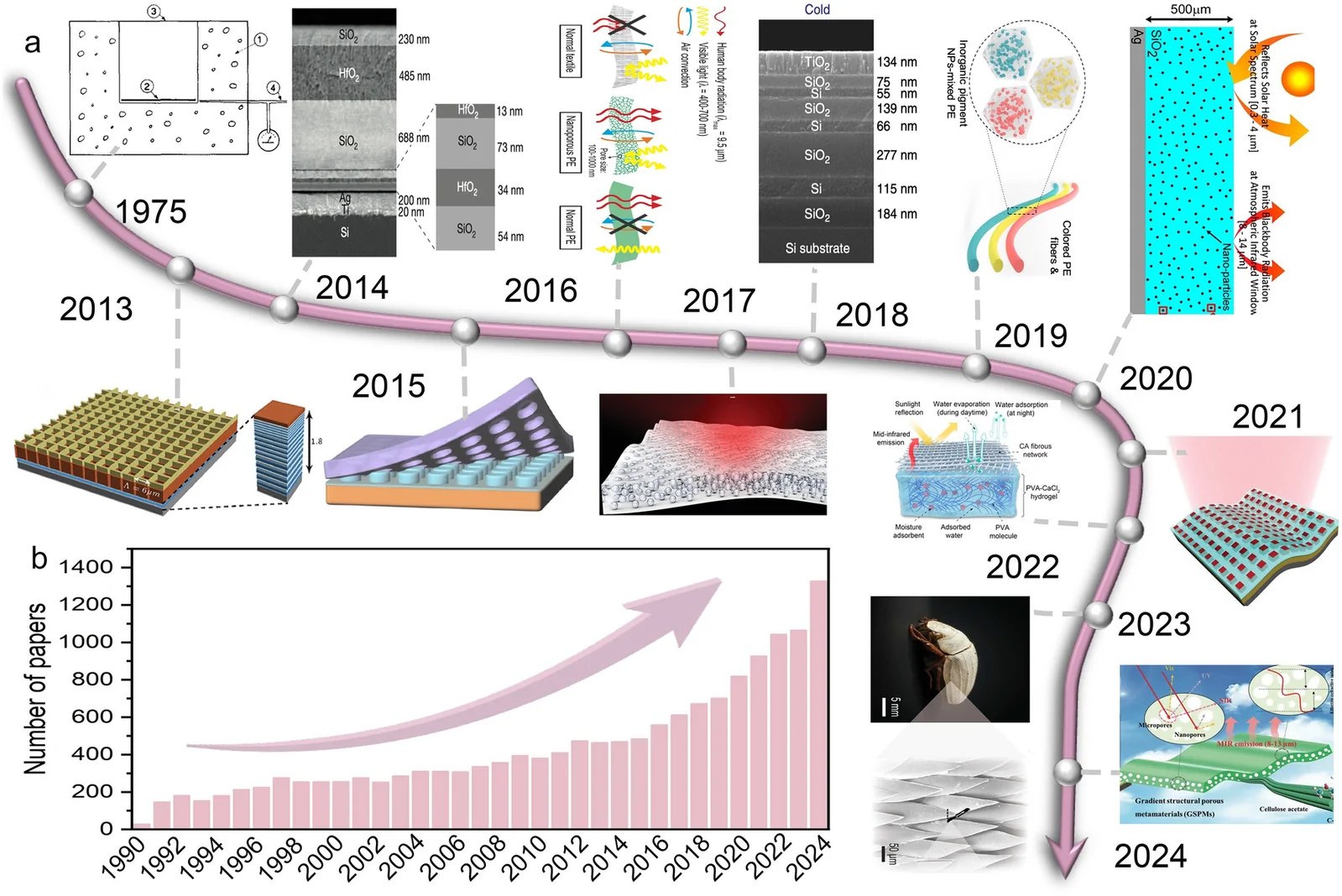
**a** Roadmap of recent advances of ERCSs. Year 1975. Reproduced with permission [35]. Copyright 1975, Elsevier. Year 2013. Reproduced with permission [40]. Copyright 2013, American Chemical Society. Year 2014. Reproduced with permission [50]. Copyright 2014, Springer Nature. Year 2015. Reproduced with permission [23]. Copyright 2015, American Chemical Society. Year 2016. Reproduced with permission [168]. Copyright 2016, The American Association for the Advancement of Science. Year 2017. Reproduced with permission [103]. Copyright 2017, The American Association for the Advancement of Science. Year 2018. Reproduced with permission [192]. Copyright 2018, Elsevier. Year 2019. Reproduced with permission [128]. Copyright 2019, Elsevier. Year 2020. Reproduced with permission [127]. Copyright 2020, American Chemical Society. Year 2021. Reproduced with permission [196]. Copyright 2021, The American Association for the Advancement of Science. Year 2022. Reproduced under the terms of CC BY 4.0 [134]. Copyright 2022, The American Association for the Advancement of Science. Year 2023. Reproduced with permission [138]. Copyright 2023, The American Association for the Advancement of Science. Year 2024. Reproduced with permission [136]. Copyright 2024, Wiley–VCH. **b** Number of publications in recent decades (Data get from Web of Science by searching the key word of radiative cooling on Dec 12th, 2024)

RCS is a promising future sustainable cooling technology that effectively conserves energy by operating without the consumption of external energy [8, 19–22]. It operates in a zero-energy-consumption way and achieves this by allowing heat to be dissipated from tailored structures through atmospheric window into outer space, where the temperature is 3 K, far colder than the Earth’s surroundings [23–27]. Typically, RCSs are commonly classified into two distinct categories: nighttime radiative cooling (NTRC) and daytime radiative cooling (DTRC) [19, 28, 29]. Both of them are enabled to efficiently release heat into the ambient environment, either by emitting a large amount of heat in the mid-infrared (MIR) range or/and by reflecting a significant portion of the solar spectrum [10, 30–33]. The prior called Arago initially proposed the concept of radiative cooling in early 1828, which indicated that emission of heat through radiation is possible from any item. Temperature of an object can be reduced as much as the heat emitted by radiation exceeds the heat received from the surroundings [26, 34]. Furthermore, NTRC has been the subject of systematic study since the 1970s, where the experimental evidence was presented to establish the practicality of lowering the temperatures of material surfaces to below the surrounding environment during nighttime via radiative cooling method [35]. A further development of engineered radiative cooling systems (ERCSs) was undertaken to expand its applicability for daytime use. It is important to acknowledge that DTRC presents a greater challenge compared to nighttime cooling approach, since the heat produced by the sun can significantly counterbalance the overall cooling effectiveness [36–39]. It was not until 2013 that it was feasible to suggest a cooling system for a structure that was designed by Rephaeli, which is capable of accomplishing a radiative cooling effect in the presence of direct sunlight [40]. In order to effectively reduce solar gain, the optimal materials for DTRC must have a high emissivity of the MIR range and a relatively low solar absorptivity [41–45]. To date, the evolution of a series of progressed structures and materials for ERCSs that can achieve efficient cooling effects is revealed in Fig. 1 [9, 12, 16, 46–50].

With rapid development of RCS, this review put more emphasis on the engineered structures and emerging applications associated with thermal-regulating and energy-saving for ERCSs, highlighting the most significant achievements [51–54]. Initially, fundamental theories are introduced, e.g., the basic concept of radiative cooling technology. Subsequently, we classify the recent advancements of engineered designs for RCSs, which include nature-inspired structures, colored materials, meta-structural configurations, and multilayered constructions. Following that, we go through primary applications associated with thermal-regulating textiles (daytime-, evaporative-, and responsive-radiative cooling) and energy-saving devices used in different scenes (e.g., buildings, wound dressing, water harvesting, electronics, photovoltaics, and generation). Additionally, individual viewpoints on prospective research and potential challenges of ERCSs in terms of scalable production and sustainable practicality are highlighted. This review offers a comprehensive introduction and analysis of ERCS, thereby acting as a crucial reference for advancing the practical and successful use of the radiative cooling technology.

### Basic Concept of ERCSs

**Fig. 2:**
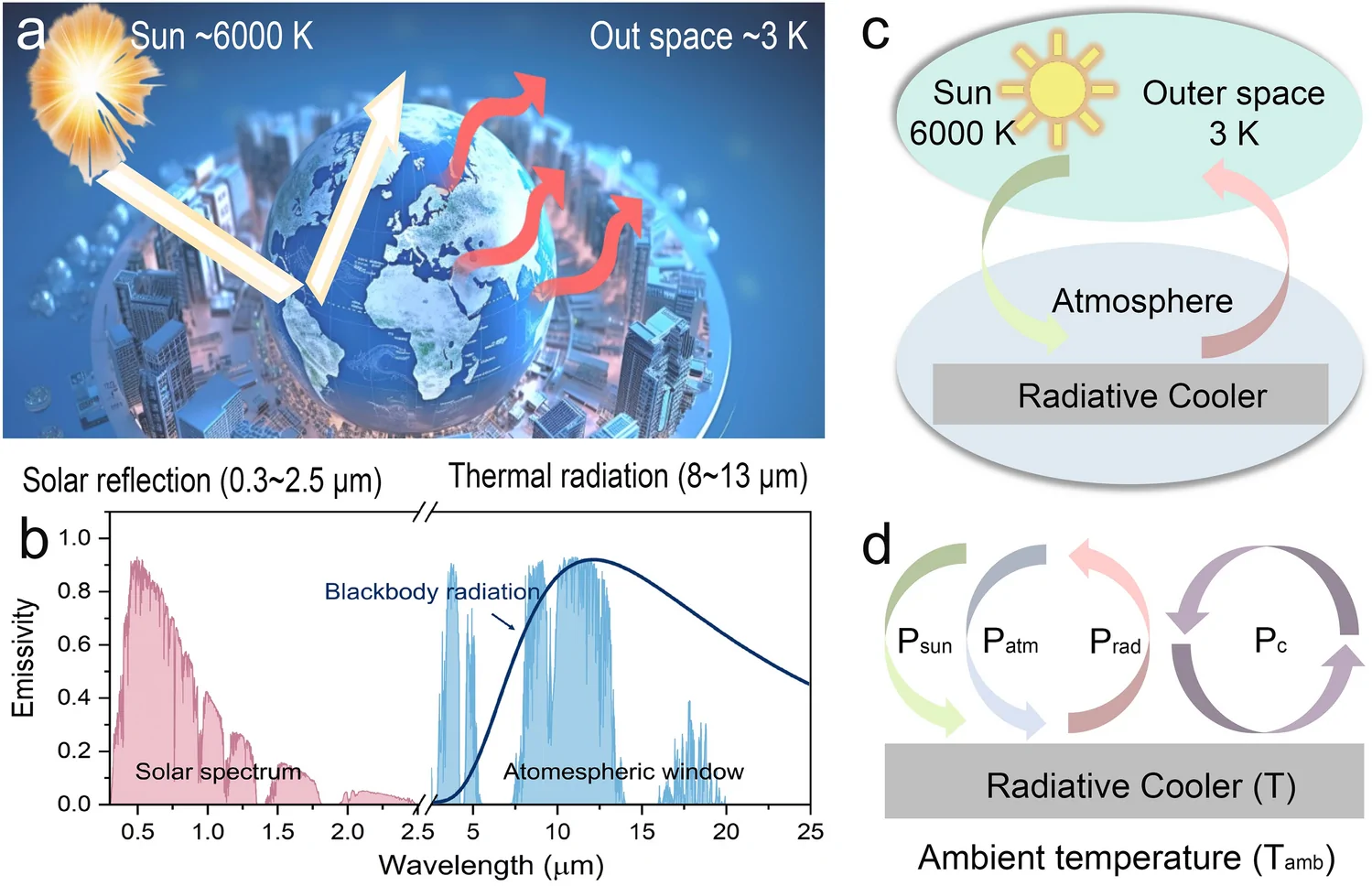
**a** Schematic of radiative heat transfer from terrestrial surfaces to outer space. **b** Radiation spectrum of the human body (black), the normalized AM 1.5 global sun spectrum (pink), and the atmospheric transmittance spectrum (blue). Reproduced with permission [42]. Copyright 2021, Elsevier. **c** Radiative heat exchange process of the ERCS. **d** Thermal energy transmission in a conventional ERCS. *P*_sun_ represents the energy absorbed by the radiative cooler. *P*_amb_ (*T*_amb_) denotes the power absorbed by the radiative cooler from atmospheric radiation. Pc represents the nonradiative energy derived from the atmosphere. *P*_rad_ (*T*) denotes the thermal radiative power of the radiative cooler

The second law of thermodynamics states that heat will naturally flow from a high-temperature item to a low-temperature one, and this transformation is irrevocable. Outer space is an immense cold reservoir, with a temperature of merely 3 K, which significantly lower than that of any terrestrial object [55–58] (Fig. 2a). Consequently, the object can autonomously release heat as thermal radiation into outer space, leading to a decrease in its temperature and facilitating radiative cooling effect [59, 60]. The Earth’s atmospheric system typically maintains an equilibrium circulation of energy, as the radiation that escapes to outer space equilibrates with the approaching solar energy [61, 62]. The majority of short-wave (0.3–2.5 µm) solar spectrum radiation that enters the atmosphere is absorbed by terrestrial objects, while the remaining portion is reflected back to the outer space [63–65]. Parallelly, terrestrial objects release/emit thermal radiation within the long wavelength spectrum, specifically ranging from 2.5 µm to 50 µm [66, 67]. Within this range, the majority of radiation is absorbed, while a portion is able to radiate directly into outer space through the atmospheric transparent window (ATW) in the region of 8–13 µm [68–70]. The cooling effect of ERCSs can be realized, an object absorbs solar heat gain that is less than the amount it radiates into outer space, when it exposed to the atmosphere on the Earth [71, 72] (Fig. 2b). The core principle of the ERCSs for efficient net cooling is to accurately regulate the optical properties across the extensive thermal radiative spectrum from ultraviolet region to infrared wavelength, hence optimizing radiative energy loss to outer space and reducing solar heat absorption [73–76].

Investigating the energy dynamics of a radiative cooler of a unit area subjected to the atmospheric scenario, which is devoted to the net cooling power (denoted as P_cool_). This phenomenon occurs when the outgoing energy exceeds the incoming heat, indicated as *P*_out_ and *P*_in_, respectively. This relationship can be exhibited as follows [38, 49, 77–79]:

1 $$P_{{{\mathrm{cool}}}} \left( T \right) = P_{{{\mathrm{out}}}} - P_{{{\mathrm{in}}}}$$

2 $$P_{{{\mathrm{out}}}} = P_{{{\mathrm{rad}}}} \left( T \right)$$

3 $$P_{{{\mathrm{in}}}} = P_{{{\mathrm{sun}}}} + P_{{{\mathrm{atm}}}} \left( {T_{{{\mathrm{amb}}}} } \right) + P_{{\mathrm{c}}} \left( {T, T_{{{\mathrm{amb}}}} } \right)$$

where *P*_rad_ denotes the thermal radiation emitted from the radiative cooler, the *P*_sun_ represents absorbed solar irradiation by the radiative cooler, the *P*_atm_ indicates the absorbed energy from the atmosphere by the radiative cooler, and the P_c_ refers to the nonradiative energy including conduction and convection. The *T*_amb_ represents the absolute ambient temperature, while T is the absolute temperature of the radiative cooler [19, 28, 80].

To further elucidate the fundamental principles associated with ERCSs, we shall initially examine the case of daytime radiative coolers. A daytime radiative cooler, when positioned to access a clear sky, is capable of achieving a temperature below the ambient atmosphere, even in the presence of direct sunlight [14, 16, 81]. In this scenario, we believe that the radiative cooler is facing the sky, which is characterized by an area A and a temperature T, and orientated zenith direction (Fig. 2c). Thus, the net cooling power of a daytime radiative cooler, denoted as P_cool_, can be defined as follows [22, 33, 82, 83]:

4 $$P_{{{\mathrm{cool}}}} \left( T \right) = P_{{{\mathrm{rad}}}} \left( T \right) - P_{{{\mathrm{sun}}}} - P_{{{\mathrm{atm}}}} \left( {T_{{{\mathrm{amb}}}} } \right), - P_{{\mathrm{c}}} \left( {T, T_{{{\mathrm{amb}}}} } \right)$$

In the aforementioned equations, *P*_rad_, *P*_sun_, and *P*_atm_ are delineated according to Planck’s blackbody radiation law as follows [14, 84]:

5 $$P_{{{\mathrm{rad}}}} \left( T \right) = A\int d{\Omega }\cos \theta \mathop \int \limits_{0}^{\infty } \varepsilon \left( {\lambda ,\theta } \right)I_{bb} \left( {T,\lambda } \right){\mathrm{d}}\lambda$$

6 $$P_{{{\mathrm{sun}}}} = A\mathop \int \limits_{0}^{\infty } {\mathrm{d}}\lambda \varepsilon \left( {\lambda ,\theta_{\lambda } } \right)I_{{{\mathrm{sun}}}} \left( \lambda \right)$$

7 $$P_{{{\mathrm{atm}}}} \left( {T_{{{\mathrm{amb}}}} } \right) = A\int {{\mathrm{d}}\Omega \cos \theta } \int\limits_{0}^{\infty } {\varepsilon_{{{\mathrm{atm}}}} \left( {\lambda ,\theta } \right)I_{bb} \left( {T_{{{\mathrm{amb}}}} ,\lambda } \right){\mathrm{d}}\lambda }$$

8 $$P_{{\mathrm{c}}} \left( {T,T_{{{\mathrm{amb}}}} } \right) = h_{{\mathrm{c}}} \left( {T_{{{\mathrm{amb}}}} - T} \right)$$

$\int {{\mathrm{d}}\Omega } = \int\limits_{0}^{\pi /2} {{\mathrm{d}}\theta \sin \theta } \int\limits_{0}^{2\pi } {{\mathrm{d}}\phi } \mathop \int \limits_{0}^{2\pi }$ is the angular integral across a hemisphere, while *I*_bb_(*T*, *λ*) represents the blackbody radiance at a specific temperature and wavelength, determined by $I_{bb} = \frac{{2hc^{2} }}{{\lambda^{5} }}\frac{1}{{e^{hc/\lambda \kappa T} - 1}}$ where h denotes the Planck constant, κ represents the Boltzmann constant, c signifies the speed of light, and λ indicates the wavelength. In Eq. (5), T represents the surface temperature of the radiative cooler. $\varepsilon \left( {\theta ,\lambda } \right)$ denotes the emissivity of the radiative cooler. Similarly, $\varepsilon \left( {\theta_{\lambda } ,\lambda } \right)$ is the emissivity or absorptivity of the radiative cooler within the solar spectrum, and *I*_sun_(λ) signifies the direct spectral solar irradiance in Eq. (6). Meanwhile, *T*_amb_ represents the ambient temperature of the atmosphere, and the $\varepsilon_{{{\mathrm{atm}}}} \left( {\lambda ,\theta } \right)$ denotes the emissivity of the atmosphere in Eq. (7), influenced by several parameters (e.g., humidity, altitude, and cloud coverage). Ultimately, P_c_ is attributed to the convection and conduction in Eq. (8). Where *h*_c_ represents the overall heat transfer coefficient, expressed as *h*_c_ = *h*_conv_ + *h*_cond_, denoting the heat transfer coefficients for convection and conduction, respectively (Fig. 2d).

Given the aforementioned facts, it is important to note that the optical properties (r and ε) of radiative cooler in the solar spectrum and MIR range, respectively, governing radiative heat exchange between a radiative cooler and the atmosphere [13, 85]. Thus, we primarily concentrate on the impact of solar reflectivity and MIR emissivity on ERCSs, as they determine the net cooling effect of total heat energy exchange from radiative coolers [86, 87]. To optimize the radiative cooling effect, meticulous regulation of the optical characteristics of the radiative cooling materials is essential to achieve almost reflectivity across the whole solar spectrum of 0.3–2.5 µm, alongside total thermal radiation through the ATW or high infrared emissivity in the MIR range of 8–13 µm [88].

Mechanism of ERCSs dynamically controls thermal management by adjusting solar reflectivity and MIR emissivity. Solar reflectivity is regulated via reversible electrochemical processes in electrochromic materials, which modify their optical characteristics within the solar spectrum (0.3–2.5 µm), facilitating transitions between transparent and reflective states to control solar heat gain [89, 90]. Simultaneously, MIR emissivity, essential for radiative heat dissipation in the atmospheric window (8–13 µm), is modified by altering surface properties or integrating emissive layers with adjustable molecular vibrations. Advanced methodologies like as meta-structure and the use of phase-change materials significantly improve this modulation, enabling ERCSs to adaptively regulate cooling and heating requirements [91, 92]. Design challenges arise from trade-offs: high solar reflectivity may inhibit thermal emission, while significant infrared absorption poses a risk of thermal accumulation in the absence of sufficient emission. Advanced nanostructured coatings and multilayer photonic systems address these challenges by enhancing spectral selectivity; however, inherent physical limitations necessitate strategic compromises. Application-specific material design is essential for optimizing solar reflectance, infrared absorption, and emissive performance, facilitating energy-efficient thermal equilibrium and promoting the development of next-generation passive cooling technologies [93]. Collectively, these processes offer a flexible method for dynamic temperature management, supporting the functional complexity of ERCSs in energy-efficient building and thermoregulatory textiles.

## Designed Materials and Structures of ERCSs

**Fig. 3:**
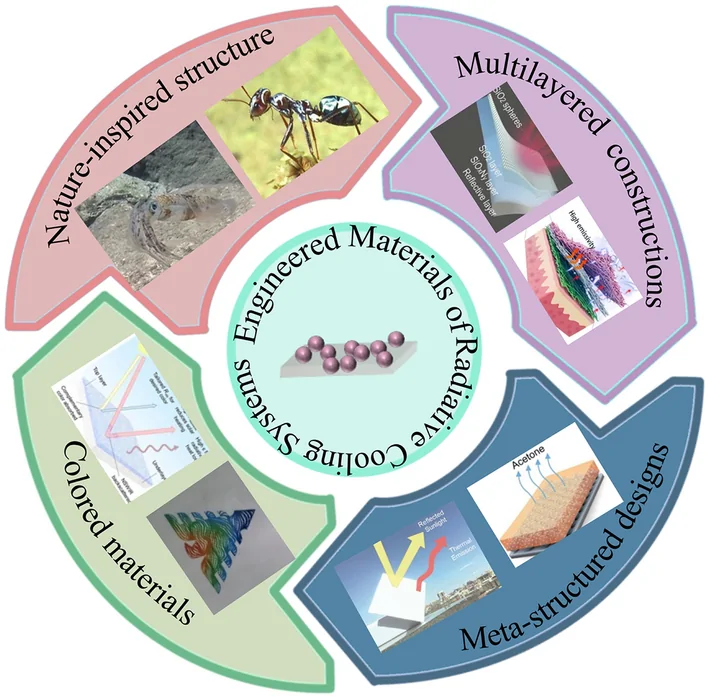
Engineered materials of radiative cooling systems, including nature-inspired structure, colored materials, meta-structured designs, and multilayered constructions. Reproduced with permission [113]. Copyright 2015, The American Association for the Advancement of Science. Reproduced with permission [116]. Copyright 2018, The American Association for the Advancement of Science. Reproduced under the terms of CC-BY 4.0 license [131]. Copyright 2020, The American Association for the Advancement of Science. Reproduced under the terms of CC BY license [126]. Copyright 2022, Springer Nature. Reproduced under the terms of the CC BY license [146]. Copyright 2021, Springer Nature. Reproduced with permission [142]. Copyright 2018, The American Association for the Advancement of Science. Reproduced under the terms of CC-BY license [156]. Copyright 2023, Springer Nature. Reproduced with permission [160]. Copyright 2022, Wiley–VCH

The fundamental principles of radiative cooling demonstrate that the radiative characteristic of the cooler is a crucial factor for achieving an effective radiative cooling effect [94, 95]. Previously, natural materials and manufactured polymers were the forerunners of radiative cooling technology. Additionally, a range of energy-efficient radiators, including as colored paints and coated films, were consistently developed for NTRC. On the other hand, numerous advancements in innovative technologies, novel materials, and structures have been developed to attain the DTRC effect, encompassing nature-inspired designs, chromatic behaviors, meta-structured constructions, and multilayered structures in Fig. 3 [8, 20, 36, 48, 96–100]. The developed materials and architectures of the ERCSs were focused on certain ranges, such as the solar spectrum of 0.3–2.5 µm and the MIR spectrum of 2.5–25 µm. This section primarily summarizes, classifies, and discusses commonly utilized and advanced radiative coolers of ERCSs [101–103].

### Nature-Inspired Designs of ERCSs

Phenomena of radiative adaptation are prevalent for biological systems. In nature, organisms can possess tailor-made optical and thermoregulatory systems, particularly within the microstructures present on the surfaces of various organisms [96, 100, 104, 105]. The interaction between light and matter through these microstructures decides considerable importance for the evolution and survival of organisms [99, 106, 107]. Drawing from the fascinating characteristics of natural organisms, researchers have invested significant effects into the development of sophisticated infrared adaptive materials, while also investigating their potential uses in smart camouflage and various other infrared-related technological domains [108–110]. This review summarizes the influence of microstructures on improving the optical effects and delivering the novel spectral characteristics in biological systems, emphasizing the thermoregulatory mechanisms of solar and infrared spectra produced by these microstructures and their significance in thermal radiation. The intricate designs of these photonic structures, along with the fundamental physical principles, have sparked significant interest in the enhancement of innovative photonic materials, leading to a diverse array of nature-inspired microstructures aimed at thermal-regulating and energy-saving applications [111, 112].

**Fig. 4:**
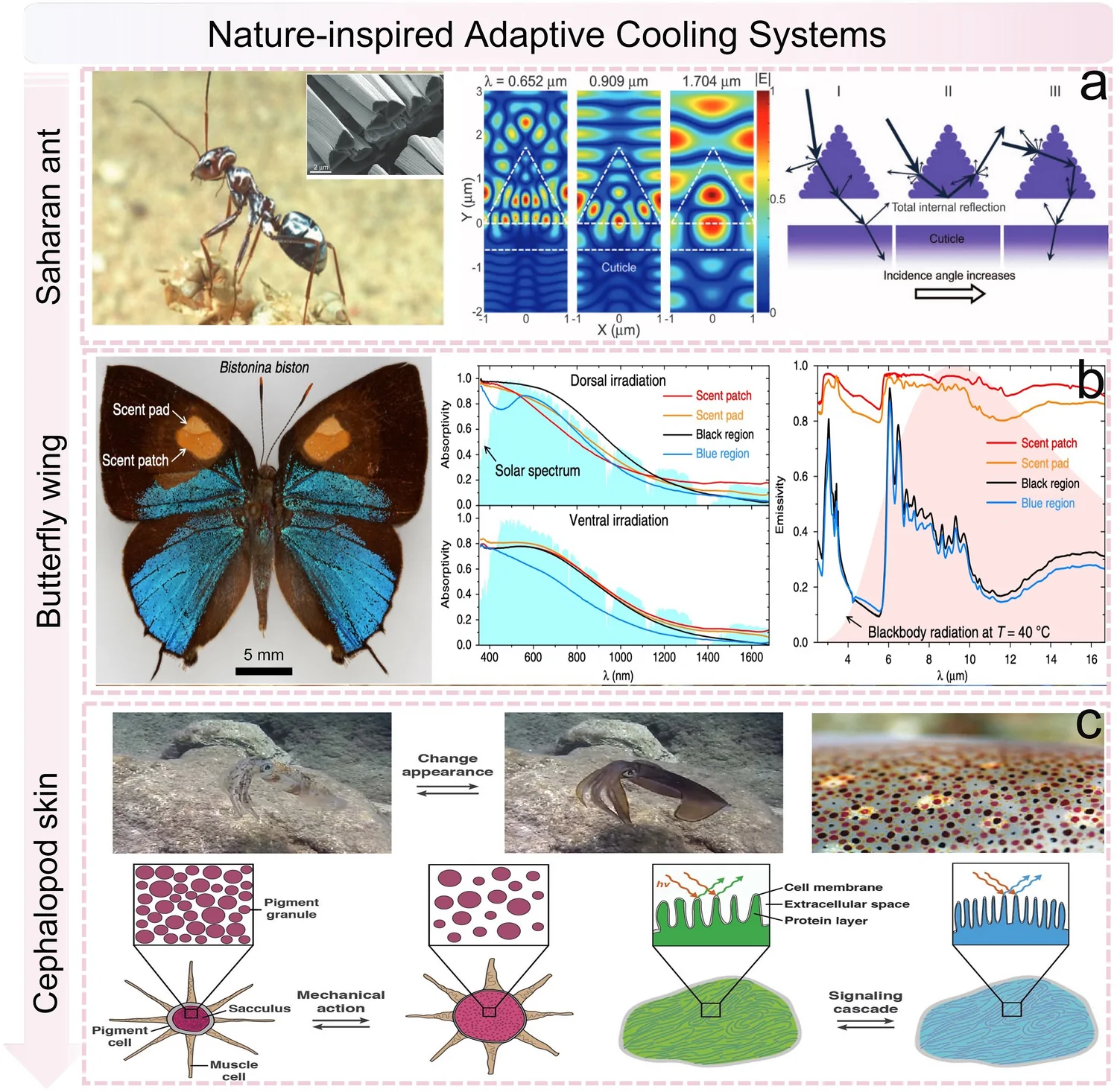
Nature-inspired adaptive cooling systems. **a** Image of a silver ant on the left; cross-sectional representation of a two-dimensional light field distribution in the middle; schematic illustrating the interaction between visible and near-infrared light on the right. Reproduced with permission [113]. Copyright 2015, The American Association for the Advancement of Science. **b** Photograph of a male butterfly, left; solar absorption spectra recorded from different regions, middle; and thermal emissivity spectra, right. Reproduced under the terms of the CC BY license [115]. Copyright 2020, Springer Nature. **c** An image of squid skin, with yellow, red, and brown chromatophores, and iridocytes, top. The schematic of a cephalopod chromatophores organ, and a squid iridocyte, bottom. Reproduced with permission [116]. Copyright 2018, The American Association for the Advancement of Science

To that purpose, Shi et al. [113] highlighted that the notable silvery appearance of the Saharan ants is attributed to a dense arrangement of triangular hairs that serve two thermoregulatory effects. One of the primary roles is to improve the reflectivity of the ant’s surface within the solar spectrum including visible and near-infrared wavelengths, where the solar radiation is most intense. Meanwhile, another role of its surface construction is to increase the emissivity of the ant in the MIR range as much as possible [113]. The latter effect endowed the animals with effectively dissipating heat to the ambient surroundings through blackbody radiation. One kind of specimen of *Cataglyphis bombycina* (Saharan ant) is exhibited in Fig. 4a (left), which was demonstrated that the dorsal and lateral aspects of the body could exhibit a silvery sheen and were adorned with thick, homogeneous arrays of hairs. Their most notable structural characteristic was the triangle cross section, distinguished by two corrugated upper facets and a flat lower facet oriented toward the ant’s body (inset in Fig. 4a, left). Basically, individual hairs of given cross-sectional dimensions generated enhanced reflection due to scattering at specific wavelengths where fundamental and higher-order Mie resonance modes were supported (Fig. 4a, middle). Due to the variation in cross-sectional areas, resonance peaks from individual hairs were averaged out, so that the hair cover effectively acted as a coating with enhanced broadband reflection [113]. Moreover, the correlation between reflectivity and incidence angle was demonstrated, revealing that reflectivity enhancement becomes significantly pronounced beyond 30° as the incidence angle increases (Fig. 4a, right, I). This was the threshold angle at which entire internal reflection initiates at the lower surfaces of the hairs (Fig. 4a, right, II). As angles approaching to 90°, the reflectivity diminished when entire internal reflection at one of the higher facets increasingly directs radiation (Fig. 4a, right, III). Conclusively, Saharan silver ants possessed a dense arrangement of triangular hairs on the dorsal and lateral surfaces of their bodies. Where the silvery hairs shielded the ants from overheating by reflecting solar spectrum and releasing MIR radiation [113].

A living structure, butterfly wing, contains a matrix of living cells whose function requires appropriate temperatures [114, 115]. Cheng-Chia Tsai1 et al. [115] analyzed the butterfly wings across a wide range of simulated environmental conditions and found out that regions containing living cells were maintained at cooler temperatures. Meanwhile, diverse size nanostructures and variable cuticle thicknesses could generate a heterogeneous distribution of radiative cooling performance, selectively lowering the temperature of structures like wing veins and androconial organs. The male Bistonina biston is a widely recognized ecological creature, found globally, participates in long-distance migration, and exhibited strong polyphagy [115] (Fig. 4b, left). The wing features androconial organs, which includes a “scent patch” and a “scent pad,” both of which are wrapped up by modified scales. Spectroscopic evaluations indicated that all wing zones exhibited significantly decreased solar absorptivity in the near-infrared wavelength from 0.7 to 1.7 µm compared to the visible spectrum (Fig. 4b, middle). This result contributed to a reduction in wing temperature, since these areas were more prone to overheating from solar exposure than bulkier portions of the body [115]. Furthermore, they performed hyperspectral imaging of the butterfly wings in the MIR range of 2.5–17 µm and discovered that various regions of the butterfly wing exhibited significantly variable thermal emissivity. Where the scent patch, scent pad, and wing veins displayed emissivity approximately unity, indicative of a perfect blackbody. Behavioral experiments indicated that butterflies utilized their wings to detect visible and infrared radiation, exhibiting unique actions to mitigate wing overheating. This work emphasizes the physiological significance of wing temperature and its precise regulation through structural and behavioral adaptations [115].

To develop adaptive variants, Xu et al. [116] announced the adaptive infrared-reflecting frameworks that possessed a simple actuation mechanism, rapid reaction, self-governing functionality, and facile manufacturability, inspired by cephalopod skin. The skin of cephalopods serves as a compelling source of inspiration due to its dynamically color-responsive capabilities. The pigmentation and arrangement of cephalopod skin could be modified independently and periodically for camouflage or communication for the specimen of squid [116, 117]. The impressive capabilities of camouflage were facilitated by the intricate structure of the squid’s skin, which houses innervated dermal layers containing chromatophore pigment cells and reflective cells identified as iridocytes (Fig. 4c, top). Radial muscle cells expanded and contracted internal sacculi that were filled with pigment granules in adaptive chromatophore pigment cells. These yellow, red, and brown organelles functioned as size-variable biological spectrum filtration systems, absorbing and reflecting specific wavelengths of visible light. A biological conveying cascade could alter the configurations and refractive index disparities of responsive iridocytes, attributed to its alternated arrangements of membrane-bound nanostructured protein layers and extracellular space (Fig. 4c, bottom). Their discoveries enable to facilitate advancements in infrared camouflage and other devices that manage infrared radiation [116].

### Colored Materials of ERCSs

Cooling terrestrial items, including buildings, vehicles, and textiles, presents a significant difficulty in contemporary society. However, cooling is frequently accomplished by compression-based systems that necessitate substantial energy consumption. Consequently, alternative strategies, associated with minimal energy expenditure and a net cooling impact, are preferred [118–120]. The manufactured materials have been shown to produce excellent radiative cooling effect by passively regulating solar absorption and MIR radiation, displaying elevated reflectance in the solar spectrum and significant emissivity in the MIR range [121, 122]. Typically, the majority of materials suggested for DTRC are developed to optimize radiative cooling by the utilization of metallic mirrors or highly reflective white substances across the entire solar spectrum [123, 124]. Nonetheless, their broadband reflectance in the visible spectrum limits its applicability in practical scenarios. The color combination is also a significant factor in the apparel business, presenting a major challenge to the practical application of colored materials for effective radiative cooling effects [125–127]. Researchers have explored colored materials of ERCSs to attain high solar reflectance across solar spectrum, including pigment-embedded PE, bilayer CRC paint coatings, colored CNC films, and colored CA nanofibers.

**Fig. 5:**
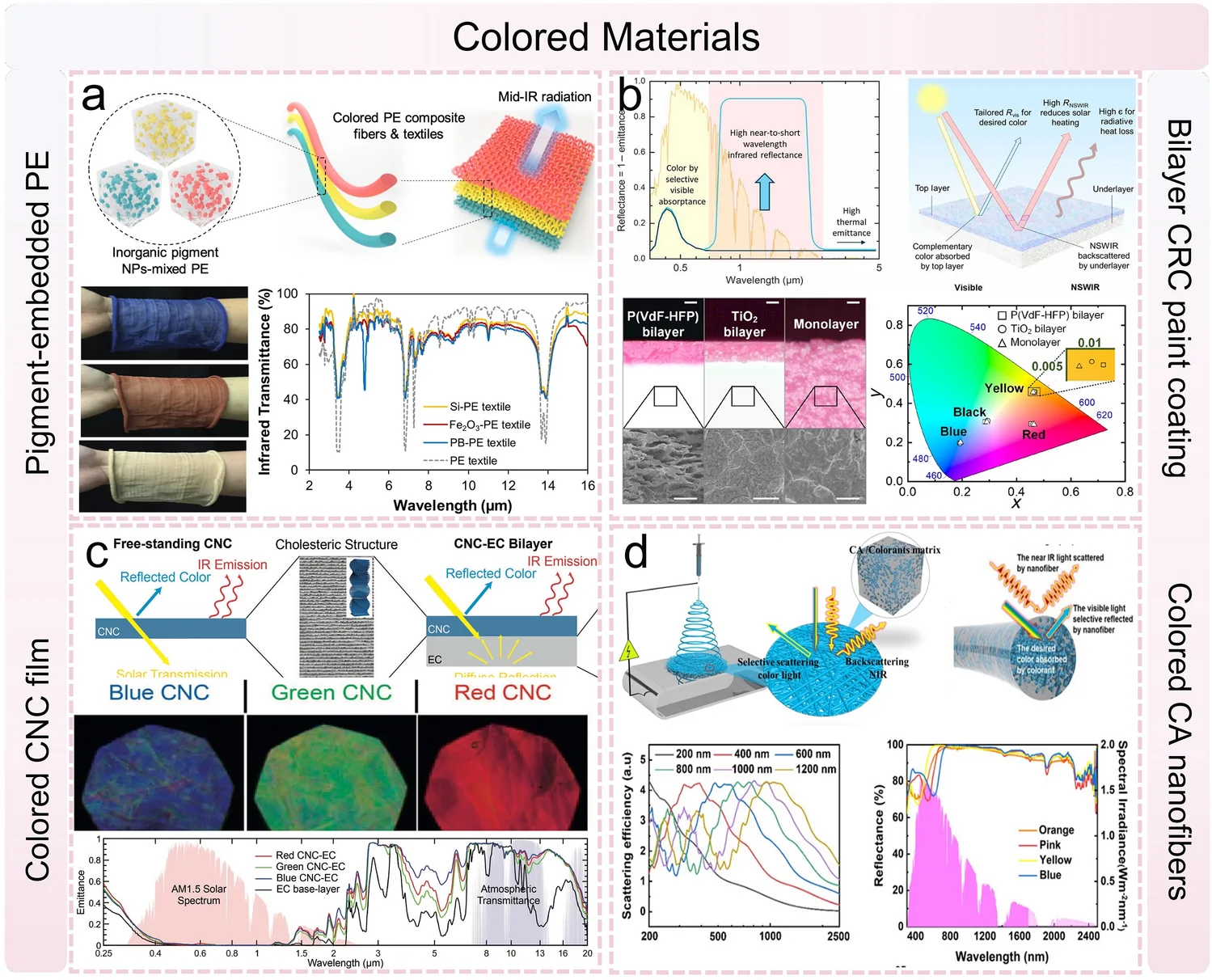
Colored materials of ERCS. **a** Pigment-embedded PE. Reproduced with permission [128]. Copyright 2019, Elsevier. **b** Bilayer CRC paint coating. Reproduced under the terms of CC-BY 4.0 license [131]. Copyright 2020, The American Association for the Advancement of Science. **c** Colored CNC film. Reproduced with permission [132]. Copyright 2022, Wiley–VCH. **d** Colored CA nanofibers. Reproduced with permission [133]. Copyright 2023, The Royal Society of Chemistry

Taking into account the difficulties of efficiently managing infrared properties of textiles while concurrently regulating their visible color, Cai et al. [128] introduced a novel technique employing inorganic nanoparticles as a chromatic element for the production of brilliantly colored, infrared-transparent textiles, as demonstrated in Fig. 5a. This pigment-embedded polyethylene (PE) was produced by effectively identifying and employing distinctive inorganic pigment nanoparticles (e.g., Prussian blue, iron oxide, and silicon). The selection of these nanoparticles was due to their low absorption in the infrared spectrum and high reflection of certain visible wavelengths [128]. Moreover, the knitted fabrics extruded by colored PE composite exhibited a superior infrared transparency of 80% and effective radiative cooling efficacy ranging from 1.6 to 1.8 °C, alongside excellent color stability after over 100 washing cycles. This study offers a prospective resolution to the conflict between visible and infrared optical characteristics, establishing a basis for the esthetically thermoregulatory textiles [128].

The colored radiative cooler should be designed to exhibit particular optical characteristics, enabling selective absorption of specific segments of the visible spectrum (0.4 to 0.74 µm) to manifest the desired color, while reflecting other solar spectrums, especially in the near-to-short wavelength infrared (0.74 to 2.5 µm) [129, 130]. Thus, Chen et al. [131] presented a paintable two-layer coating that facilitates both coloration and radiative cooling properties, fulfilling the demand for color on solar-reflective and thermally emissive surfaces (Fig. 5b). The bilayer CRC paint coating consisted of a top layer containing a colorant and an underlayer made of porous poly(vinylidene fluoride-co-hexafluoropropene) [P(VdFHFP)] or a TiO_2_/polymer composite paint. Compared to commercial monolayer paints, the two-bilayer paint displayed almost identical colors while showing remarkably improved reflectivity in the near-to-short wavelength infrared region [131]. The visible spectra for the mono- and bilayers of each color were perfectly aligned, yielding similar CIE x and y chromaticity values with little differences in brightness. Also, the bilayer CRC paint exhibits enhanced NSWIR reflectance (by 0.1 to 0.51) compared to commercial paint monolayers of the same color and sustained a cooler temperature by 3.0 to 15.6 °C under bright sunlight [131].

To develop a scalable and sustainable cooler for structural coloration, Zhu et al. [132] introduced a vibrant, structurally colored film made from naturally sourced cellulose nanocrystals (CNCs), demonstrating an effective sub-ambient radiative cooling effect (Daytime: − 4 °C, Nighttime: − 11 °C), as illustrated in Fig. 5c. The photonic CNC films integrated the intrinsic characteristics of cellulose, such as robust MIR emittance and minimal absorption throughout the solar spectrum, allowing for customizable responses across the complete visible spectrum. The film, derived from the foundational photonic nanostructure, selectively reflected visible light, producing vibrant, fade-resistant colors while exhibiting minimal solar absorption. Coating CNC films onto a highly scattering, porous ethylcellulose (EC) base layer enabled sunlight that penetrated the CNC layer to be backscattered by the underlying EC layer, resulting in simultaneous broadband solar reflection and vibrant structural color [132]. Another strategy was demonstrated to achieve selective spectral absorption of the daytime radiative cooler. Li et al. [133] described a doped-dyeing electrospinning method to produce colored CA nanofibers, as illustrated in Fig. 5d. The colored CA nanofibers provided the selective absorption of specific wavelengths within the visible spectrum, while their nanofiber architecture ensured significant scattering of visible and near-infrared light to reduce solar heating.

### Metastructured Constructions of ERCSs

The evolution of cooling materials and structures has been significant, transitioning from photonic designs to expansive, scalable porous cooling solutions, thereby enhancing the commercial viability of this passive radiative technology. Prior research has examined the inherent thermal radiation characteristics for numerous materials [134, 135]. For instance, multilayer inorganic coatings, microporous polymer films, and plastic fabrics, enhanced infrared emissivity by the physical engineering of metastructured constructions (e.g., nano- and microstructures). Drawing from the groundbreaking studies, metastructured constructions and their related coolers have progressively developed, primarily due to their exceptional ability to manage solar radiation and MIR light simultaneously [136, 137]. Generally, metastructured constructions should exhibit the capability to achieve significant reflectivity within the solar spectrum through Mie scattering. Also, they present considerable promise in regulating MIR emission within ATW, a region that closely coincides with the thermal radiation emitted by the human body [138–140]. Given the rapid progress of ERCSs, we presented the state-of-the-art radiative coolers enabled by metastructured constructions.

**Fig. 6:**
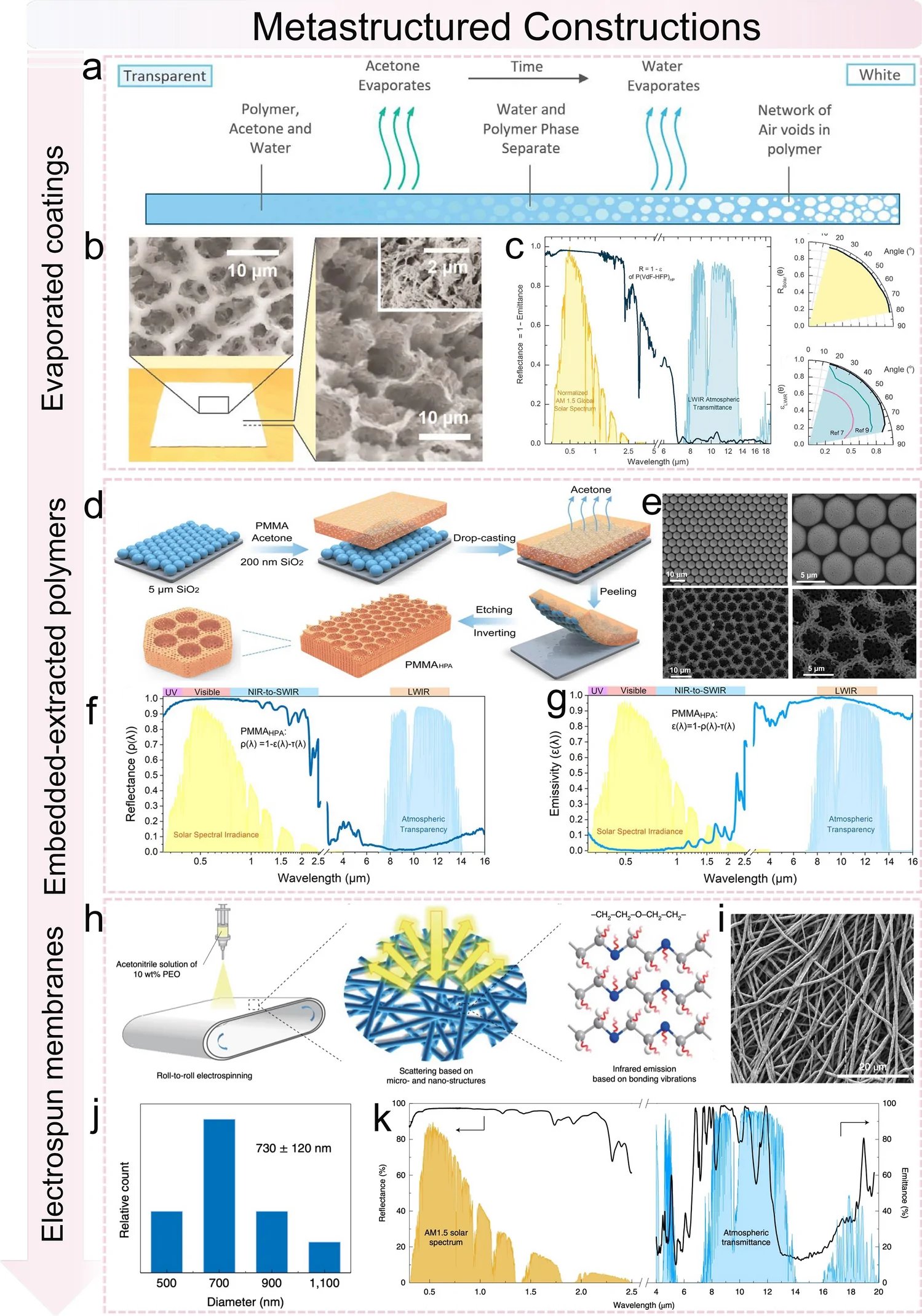
Metastructured constructions. **a** Hierarchically porous polymer coatings via phase-evaporated method. **b** Micrographs depicting top and cross-sectional views and inserted nanoporous features of P(VdF-HFP) HP. **c** Spectral reflectance of evaporated coating. Reproduced with permission [142]. Copyright 2018, The American Association for the Advancement of Science. **d** Schematic representation of the production of PMMA_HPA_ featuring a hierarchically porous structure. **e** SEM images of PMMA/SiO_2_ composite. **f** Spectral reflectance and **g** Emissivity of the PMMA_HPA_. Reproduced under the terms of the CC BY license [146]. Copyright 2021, Springer Nature. **h** Schematic of the scalable fabrication process of electrospun membrane as a selective emitter. **i** SEM image and **j** statistical distribution of the diameters of electrospun fibers. **k** Reflectivity/emissivity in the solar spectra and infrared wavelength of electrospun membrane. Reproduced with permission [149]. Copyright 2021, Springer Nature

A variety of metastructured materials can be found in nature or created through straightforward processing. These materials involve in passively cooling a surface, achieving by sunlight reflection and heat radiation to the outer space of ERCSs [141]. For instance, Mandal et al. [142] presented the evaporated coatings through a straightforward, cost-effective, and scalable phase inversion-based technique for producing hierarchically porous poly(vinylidene fluoride-co-hexafluoropropene) [P(VdF-HFP)HP] coatings that exhibited remarkable radiative cooling effects. Specifically, the phase inversion-based technique for fabricating metastructured porous polymers commenced with the formulation of a precursor solution comprising P(VdFHFP) (polymer) and water (nonsolvent) in acetone (solvent), as displayed in Fig. 6a. The metastructured constructions facilitated the swift evaporation of volatile acetone, resulting in the P(VdF-HFP) to undergo phase separation from the water. Consequently, the P(VdF-HFP)HP evaporated coating was established following the evaporation of water in Fig. 6b. The metastructured formations created by micro- and nanopores in the evaporated coating effectively backscatter sunlight and improve thermal emittance [142]. Correspondingly, the synthesized metaporous polymer, PVDF-HFP, with ~ 50% porosity and thickness ≳ 300 mm, attained a reflectivity of 96% in the sun spectrum and an emissivity of 97% in the MIR range. The elevated $\overline{R}_{{{\mathrm{solar}}}} \left( \theta \right)$ guaranteed superior sunlight reflection across all angles and obviated the necessity for silver reflectors. Concurrently, the elevated $\overline{\varepsilon }_{{{\mathrm{LWIR}}}} \left( \theta \right)$ resulted in a hemispherical $\overline{\varepsilon }_{{{\mathrm{LWIR}}}}$ that exceeded previously documented values by over 10%, as demonstrated in Fig. 6c. The precursor’s paint-like adaptability rendered P(VdFHFP) HP appealing for practical applications [142].

Regulating the total solar spectrum and MIR radiation is crucial for achieving superior cooling efficiency. Despite the significant advancements in ERCSs facilitated by metastructured cooling materials derived from evaporated porous coatings, there are still limitations in creating precise pore on the surface of evaporated coatings [143–145]. To balance the trade-off the porous distribution and cooling effect, embedded-extracted method could be utilized to create customizable porous polymers with adjustable pore structures, which theoretically facilitated cooling effects. Wang et al. [146] proposed a hierarchically oriented polymethyl methacrylate film (PMMA_HPA_) involving in a micropore array integrated with random nanopores that utilized a homogeneous and densely packed monolayer SiO_2_ microsphere template, to always achieve extraordinarily efficient radiative cooling effects.

Briefly, a mixture of PMMA and SiO_2_ nanospheres (with the size of 200 nm) in acetone was subsequently introduced into the SiO_2_ monolayer template acted as matrix, illustrated in Fig. 6d. Upon the elimination of the SiO_2_ nanospheres and the monolayer template through etching in an acid solution, one could achieve a hierarchically porous PMMA_HPA_ film characterized by arranged symmetrical micropores of ~ 4.6 μm diameter and randomized nanopores of ~ 250 nm (Fig. 6e). The optical characterizations of spectrum reflectance and emissivity of the PMMA_HPA_ film were conducted to elucidate the basis of hierarchically structured polymers for passive cooling effects [146]. The PMMA_HPA_ film, exhibiting approximately 60% porosity, demonstrated a high average solar reflectance ($\overline{\rho }_{{{\mathrm{solar}}}} = 0.95$, Fig. 6f), ensuring superior reflection from solar spectrum. Simultaneously, the PMMA_HPA_ film exhibited elevated thermal emissivity across a wide range in the MIR spectrum, continuously releasing a substantial portion of its thermal radiation even at considerable emission angles ($\overline{\varepsilon }_{{{\mathrm{LWIR}}}} = 0.98$, Fig. 6g). These properties significantly enhanced the radiative heat exchange between the cooling structural polymer and the atmospheric surroundings, achieving effectively all-day passive radiative cooling effects, due to the high $\overline{\rho }_{{{\mathrm{solar}}}}$ and $\overline{\varepsilon }_{{{\mathrm{LWIR}}}}$ of as-obtained PMMA_HPA_ film [146].

More importantly, electrospun technology provides an efficient and scalable approach for the development of cost-effective, high-performance metastructured photonic materials aimed at addressing the energy crisis and reducing the greenhouse effect [147, 148]. Li et al. [149] developed a hierarchically structured polymer nanofiber film, fabricated by a scalable electrostatic spinning technique, which facilitated selective MIR emission, efficient sunlight reflection, thus achieving effective all-day radiative cooling efficacy. The PEO films, consisted of random nanofibers, were manufactured as daytime radiative coolers using a scalable electrospinning process, as exhibited in Fig. 6h. The white color of es-PEO films indicated significant scattering of visible light, attributed to the multilayered structure of irregularly stacked nanofibers with a broad diameter distribution centered around ~ 800 nm (Fig. 6i) [149]. Based on the Mie theory, the scattering efficiency calculation of the es-PEO nanofiber confirmed that nanofibers with diameters ranging from 500 to 1,200 nm could effectively scatter sunlight, particularly within 0.3–1.2 µm wavelength range, reflecting the majority of the solar spectrum (Fig. 6j). As displayed in Fig. 6k, it illustrated the optical spectrum of the es-PEO film that exhibited a high reflectivity of 96.3% in the solar spectrum and an excellent emissivity of 78% within 8–13 µm wavelength, making it optimal for achieving superb radiative cooling performance [149]. This study offers novel pathways for the advancement of large-scale, high-performance radiative cooling technologies aimed at fostering an energy-efficient and sustainable society. Furthermore, it has the potential to stimulate more molecular and chemical designs, boosting an interdisciplinary approach for the development of enhanced metaphotonic materials toward ERCSs.

### Multilayered Structures of ERCSs

The rapid worldwide industrialization and population surge generate substantial needs for cooling across buildings, electronics, and individuals, which exacerbates the energy usage and associated environmental implications of conventional cooling methods (e.g., vapor compression and air conditioning) [150, 151]. Effective techniques, included previously described nature-inspired systems and metastructured constructs, were demonstrated to boost the radiative cooling performance [152]. Furthermore, multilayered structures were investigated for enhanced reflection in the solar spectrum and elevated emissivity in the MIR wavelength range. Based on the optical characterizations, the proposed multilayered structures can effectively attain superior cooling performance throughout the heat exchange process, overcoming inadequate radiative cooling effects [153, 154].

**Fig. 7:**
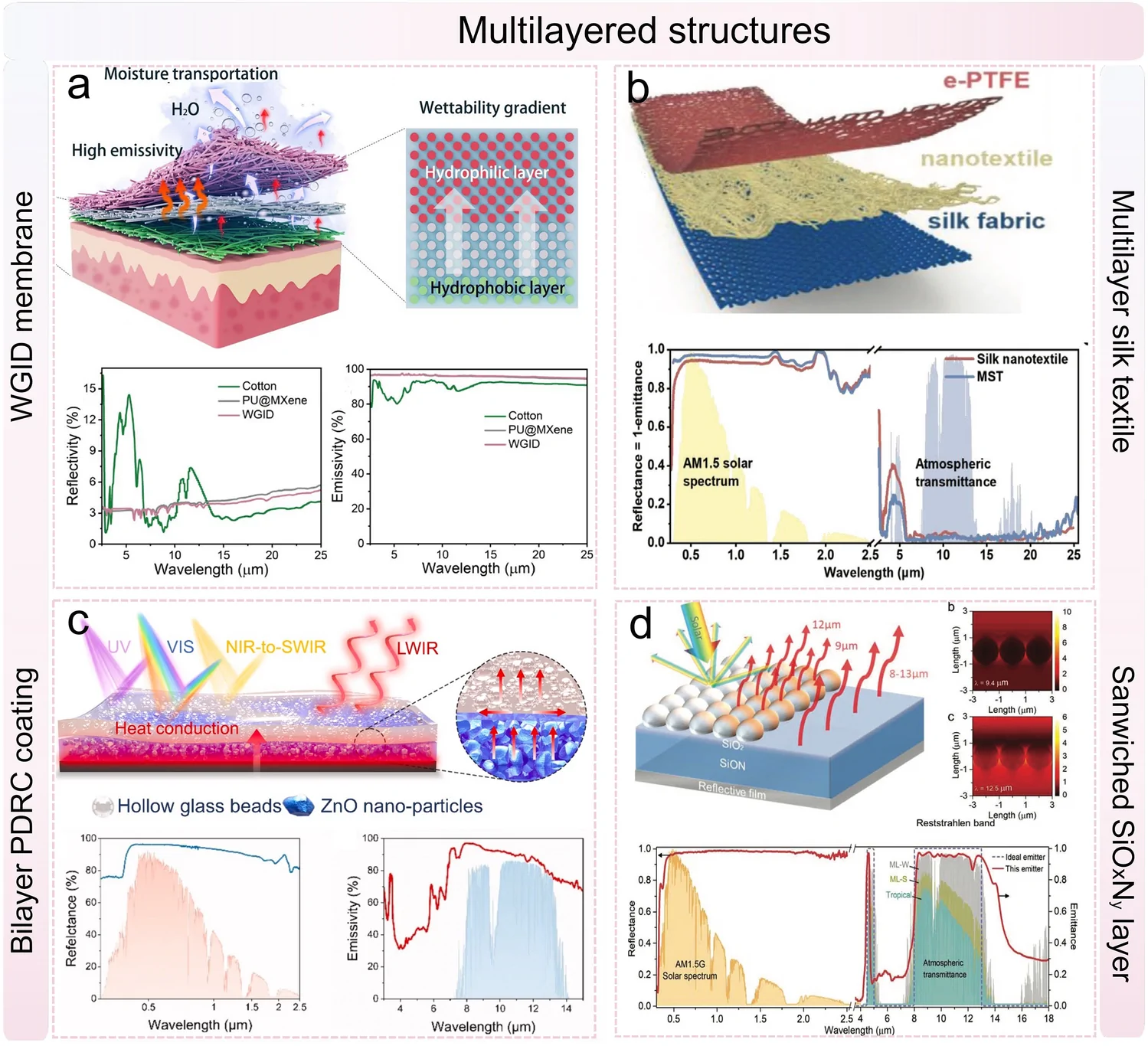
Multilayered structures of ERCSs. **a** Wettability gradient‑induced diode membrane Reproduced under the terms of the CC-BY license [156]. Copyright 2023, Springer Nature. **b** Durable radiative cooling multilayer silk textile. Reproduced with permission [157]. Copyright 2024, Wiley–VCH. **c** Structural design and optical spectra of scalable and bilayer thin PDRC film. Reproduced with permission [159]. Copyright 2024, Elsevier. **d** A solution-processed inorganic emitter with sandwiched SiO_x_N_y_ layers. Reproduced with permission [160]. Copyright 2022, Wiley–VCH

Passive radiative cooling is achieved by effectively facilitating a heat dissipation route without energy consumption. Effectively radiative cooling effect pertains to the materials with customized solar reflectivity or/and MIR emissivity [155]. Lei et al. [156] revealed a wettability-gradient-induced diode (WGID) membrane created by MXene-engineered electrospinning technique, which enhanced its heat dissipation and moisture-wicking transport. Specifically, the resultant WGID membrane achieved a cooling temperature of 1.5 °C in the “dry” state and 7.1 °C in the “wet” state, attributed to its high emissivity of 96.4% in the mid-infrared region, exceptional thermal conductivity of 0.3349 W m^−1^ K^−1^ (derived from radiation- and conduction-controlled mechanisms), as illustrated in Fig. 7a. The attained outcome primarily encompasses elevated emissivity and thermal conductivity for heat dissipation, alongside a multilayered structure with a wettability gradient for continuous moisture transport [156]. Additionally, Wu et al. [157] engineered a robust multilayer silk textile (MST) as an effective alternative to achieve enhanced radiative cooling properties without energy consumption, hence alleviating heat stress induced by global warming. This MST demonstrated exceptional overall performance, attributed to its ultrahigh solar reflectance (96.5%) and ultrahigh infrared emittance (97.1%), along with durability, air and moisture permeability. As a result, it attained an impressive sub-ambient temperature reduction of 5.1 °C under a certain of intense solar radiation, demonstrating superb passive cooling efficacy (Fig. 7b). Considering its excellent comprehensive advantages, the MST shows significant potential for practical applications in RCSs [157].

Despite the exceptional cooling efficacy of the aforementioned films, the electrospun membrane still fails to ensure the long-term stability of multilayered structures in harsh outside conditions [158]. To address this issue, Lin et al. [159] introduced an all-inorganic narrowband emitter consisting of a solution-derived SiO_*x*_N_*y*_ layer situated between a reflective substrate and a self-assembled monolayer (SiO_2_ microspheres). The resultant emitter demonstrated a high solar reflectance of 96.4% and a high infrared-selective emissivity of 94.6%, exhibiting exceptional spectral selectivity of 1.46. Exceptional subambient cooling of up to 5 °C in fall and 2.5 °C in summer was attained under high humidity conditions (Fig. 7c). This scalable synthesis method effectively enhanced the emitter’s effect, rendering it appropriate for extensive application in diverse climates. For further exploring radiative cooling applications for above ambient scenarios (e.g., vehicles, LED displays, wearable devices, etc.), the engineered coatings should demonstrate not only excellent thermal emittance and solar reflectance, but also minimal thermal resistance to efficiently dissipate significant heat flux [159]. To resolve the conflict between optimal cooling effects and thin films for practicality, Mei et al. [160] devised a bilayer thin DTRC coating, comprising a top layer of waterborne polyurethane (PU) and hollow glass beads (HGBs), and an underlayer of poly(vinylidene fluoride-co-hexafluoropropene) (P(VdF-HFP)) with zinc oxide (ZnO) nanoparticles. The configuration of DTRC coating demonstrated adequate solar reflectance (0.92), considerable infrared emissivity (0.93), and relatively high thermal conductivity (1.702 W m^−1^ K^−1^) while maintaining a minimal thickness of 100 µm, as exhibited in Fig. 7d. This innovative structural design utilizing environmentally sustainable and cost-effective bilayer thin DTRC coatings offers a novel approach for thermoregulatory systems and perhaps aids in the preservation of the Earth’s biosphere [160].

The materials and architectures of ERCSs are optimized to enhance radiative cooling through high solar reflectivity and the emission of thermal radiation within the MIR atmospheric transparency window (8–13 µm). The design of ERCS materials and structures utilizes sophisticated photonic architectures, nanocomposites, multilayer films, and colored materials to attain spectrum selectivity [13, 102]. Solar reflectivity is regulated by high-refractive-index contrast layers (e.g., TiO₂/Al₂O₃ nanocomposites) and photonic bandgap engineering to reduce solar absorption (0.3–2.5 µm) [83]. The emissivity in the MIR range (8–13 µm) is improved by the use of selective emitters, such polymers containing C–F linkages or silica-based metamaterials, as well as surface texturing to increase heat radiation. Colored materials of ERCSs signify a transformative advancement in radiative cooling, merging esthetic adaptability with energy efficiency. Utilizing fluorescence, structural photonics, and quantum-confined systems to provide subambient cooling throughout the visible spectrum [115].

Alongside the aforementioned advanced materials, Table 1 delineates the thermal features and constraints of many other designed materials utilized for radiation control-based ERCSs. Materials engineered for elevated emissivity or transmittance in the MIR region, along with strong reflectance in the sun spectrum, can effectively deliver cooling performance. Regrettably, these effective thermoregulatory materials are constrained by several restrictions, including intricate production procedures, suboptimal performance, and challenges in economic viability.

**Table 1:** Summary of materials, designs, and achievements of ERCSs

Materials	Designs/conditions	Achievements	References
Hairs of Saharan ants	Nature-inspired systems Air temperature 50 °C	High solar reflectance of 0.67, mid-infrared emissivity of 0.86, 4.3 °C decreased of the head with hairs in vacuum	[113]
Wings of butterflies	Nature-inspired system Air temperature ~ 20 or − 40 °C	High emissivity of 99.8%, ~ 6.6 °C temperature cooling	[115]
CA/CsPbX_3_ film	Colored material, Air temperature ~ 37 °C	High solar reflectivity > 90%, and MIR emissivity of ~ 95%, 2.2–5.4 °C temperature reduced	[119]
CNC film	Colored material Air temperature ~ 32 °C	Low solar absorption of 3%, over 0.9 of emissivity, sub-ambient cooling (Day: − 4 °C, Night: − 11 °C)	[132]
Cellulose acetate (CA)/nanofibers	Colored electrospun film Air temperature ~ 20 °C	Excellent solar reflectivity of 99%, and MIR emissivity of 95%, Cooling temperature of 3.2 °C	[133]
SiO_2_/TiO_2_ film	Colored material Air temperature 20 ~ 30 °C	High solar reflectivity of 87%, and MIR emissivity of 88%, Lower temperature of 9.6 °C	[126]
PVDF/CA polymer	Metastructured design Air temperature 25 ~ 30 °C	High solar reflectivity of 96%, and MIR emissivity of 96%, Reduced temperature of 4.5 °C	[143]
PU/Si_3_N_4_-FM	Metastructured design Air temperature 30 ~ 35 °C	High solar reflectivity of 91% and MIR emissivity of 93%, 2.8 °C temperature reduced	[137]
P(VdF-HFP) film	Metastructured design Air temperature 20 ~ 30 °C	High solar reflectance of 0.96 and MIR emissivity of 0.96, sub-ambient temperature drops of ~ 7 °C	[141]
PDMS-coated Al film	Metastructured design Air temperature 30 ~ 35 °C	Strong reflectivity of ∼93.4% and MIR emissivity of 94.6%, subambient cooling of ∼9.8 °C	[139]
SiO_2_/TPX	Metastructured design Air temperature 20 ~ 25 °C	High MIR emissivity of 93%, cooling power of 93 W m^−2^	[103]
PEA	Metastructured design Air temperature ~ 25 °C	High solar-reflecting of 92.2% and infrared-transparent of 79.9%, cooling up to 13 °C	[64]
CA/PVA-CaCl_2_	Multilayered structure Air temperature 30 ~ 35 °C	Great solar reflectivity of 95% and MIR emissivity of 94%, cooling temperature of 10 °C	[134]
PA/Al_2_O_3_/PA_6_	Multilayered structure Air temperature 25 ~ 30 °C	High solar reflectivity of 99%, and great emissivity of 78.13%, cooling 16.6 °C	[150]
PVDF/TEOS	Multilayered structure Air temperature 25 ~ 30 °C	Outstanding solar reflection of 97% and MIR emission of 96%, decreased temperature up to 6 °C	[152]
PVDF-HPSF film	Multilayered structure Air temperature 30 ~ 40 °C	Exceptional sunlight reflectance of 96.4% and MIR emissivity of 97.2%, reduced temperature of 6.8 °C	[154]
PDMS/HGMs/AF@Ni@PPy	Multilayer structure Air temperature 30 ~ 40 °C	Solar reflectivity of 85.5% and MIR emissivity of 95.3%, subambient cooling of 7 °C	[153]
CA@MSi_3_N_4_/PLA	Multilayered structure Air temperature 25 ~ 40 °C	Solar reflection of 99.7% and MIR radiation of 92.4%, cooling temperature of 13.8 °C	[155]
PMP/AgNWs /Wool	Multilayered structure Air temperature 35 ~ 40 °C	Solar reflection of 97% and MIR emissivity of 85.3%, cooling temperature of 8.9 °C	[177]

Typically, advanced materials of the ERCS generally exceed traditional counterparts through the incorporation of spectrum selectivity, hierarchical architectures, and metamaterial designs as displayed in Table 2. Conventional materials, including cotton, metals and glass, provide exceptional performance in either reflectivity or emissivity but do not attain adequate subambient cooling effects. Conversely, advanced materials (e.g., PMMA films, metamaterials, and dual-selective emitters) achieve over 94% reflectance within the solar spectrum and over 92% emissivity in the MIR range, facilitating efficient passive cooling effects. These innovations tackle global energy issues by providing scalable, zero-energy solutions for cooling buildings and industrial uses.

**Table 2:** Comparison of radiative emissivity among traditional materials and advanced materials

Materials type	MIR emissivity	Solar reflectivity	References
Cotton (white)	0.77–0.88	0.83	[165]
Silk	0.78	~ 0.27	[165]
Polyester	0.75–0.88	~ 0.39	[165]
Nylon	~ 0.88	N/A	[165]
Acrylic	0.81–0.88	N/A	[165]
PET	~ 0.88	~ 0.6	[109]
Aluminum	0.2–0.3	0.8–0.85	[41]
Concrete	~ 0.9	0.2–0.3	[10]
Scalable metafabric	0.945	0.924	[176]
Janus fabric	0.94	0.92	[84]
Hierarchical PMMA films	0.98	0.95	[146]
Glass-polymer metamaterial	0.93	0.96	[103]
Dual-selective emitters	0.95	0.95	[94]
Hierarchical alumina ceramic	0.965	0.996	[138]
Hybrid membrane radiator	0.96	0.97	[152]
Multilayer silk	0.971	0.965	[157]
Dual-mode TPU/PDMS fabric	0.975	0.9	[167]
Spectrally engineered textile	0.853	0.97	[177]
Sandwich-structured fabric	0.963	0.934	[183]
Temperature-adaptive textile	0.97	0.9	[185]
PA6/silk bilayer fabric	0.94	0.96	[201]

The selection of materials and constructions for ERCSs involves in a complicated interplay of application-specific demands, costs, and performance efficacy. Polymers are characterized by their cost-effectiveness and adaptability, making them appropriate for a wide range of applications, like buildings demonstrating superior cooling efficiency. In contrast, metals and ceramics possess superior durability and heat conductivity, rendering them appropriate for critical applications such as spacecraft, but at a higher expense. Nanomaterials, despite their potential, have obstacles in scalability and cost-efficiency. Layered structures, textured surfaces, and hierarchical designs each provide unique benefits to enhance cooling performance. The ideal choice is contingent upon specific requirements, such as mitigating urban heat, supporting space missions, or delivering portable cooling solutions. The chosen systems that integrating various materials and constructions are emerging as a novel solution for enhanced effects across diverse situations. Table 3 outlines the trade-offs, encompassing cooling efficiency, production costs, durability, and typical applications, thus offering a conclusive foundation for decision-making.

**Table 3:** Comparison among different materials and constructions of ERCSs across various aspects

Description	Cooling effect	Manufacturing and cost	Typical applications	Testing conditions	References
Polymers	ΔT ≈ 6–11 °C	Low complexity; low cost	Building coatings, portable cooling, sunscreen	Field tests critical for real-world	[95]
Metals	ΔT ≈ 5–10 °C	Moderate complexity; high cost	Reflective layers, heat-spreaders, spacecraft	Lab and field for durability	[100]
Ceramics	ΔT > 12 °C	High complexity; high cost	Harsh-environment installations, long-term roofs	Lab for stability, field for use	[96]
Nanomaterials	Variable; tunable emissivity	High complexity; high cost	Advanced electronics, aerospace	Lab-focused, field for scalability	[15]
Layered structures	Customizable, e.g., 5 °C sub-ambient	High complexity; high cost	Custom coatings, tailored environmental control	Lab for performance, field for use	[150]
Textured surfaces	+ 10–20% (via increased emissive area)	Moderate complexity; moderate cost	Enhanced-efficiency panels, urban surfaces	Field for environmental impact	[99]
Hierarchical designs	ΔT 6–12 °C	High complexity; high cost	Roof membranes, façade panels, specialty fabrics	Field for use, lab for performance	[75]

## Thermal-Regulating Textiles of ERCSs

**Fig. 8:**
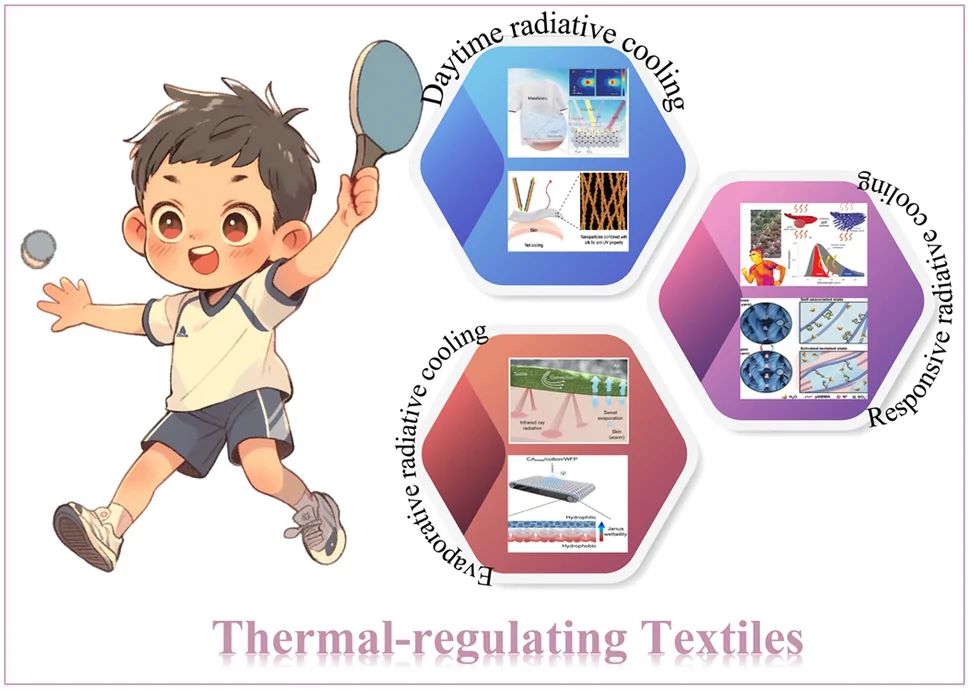
Schematic of thermal-regulating textiles, including daytime, evaporative, and responsive-radiative cooling textiles. Reproduced with permission [175]. Copyright 2021, Springer Nature. Reproduced with permission [176]. Copyright 2021, The American Association for the Advancement of Science. Reproduced with permission [182]. Copyright 2021, Elsevier. Reproduced with permission [183]. Copyright 2022, Elsevier. Reproduced with permission [187]. Copyright 2019, The American Association for the Advancement of Science. Reproduced with permission [188]. Copyright 2023, Wiley–VCH

Textiles have been essential to the development of humanity, contributing to temperature regulation and serving significant cultural functions. Meanwhile, textiles work as a medium for designers aiming to enhance apparel demand through the introduction of innovative materials and advancement of fashion [161–163]. Conventional clothing fails to effectively allow heat dissipation from the human body in hot environments, resulting in increased skin temperature [13, 164]. Thermoregulatory clothing presents a viable alternative to centralized space thermal management systems, such as heating, ventilation, and air-conditioning (HVAC) systems, due to its capacity to improve individual adaptability across various outdoor conditions [160, 164]. The textiles designed for thermal regulating applications, showcasing exceptional characteristics like unique optical scattering and remarkable wearability, have been produced through three main methods [165, 166]: (1) the use of coatings and lamination techniques for functional nanoparticles on textile surfaces; (2) the process of electrospinning to incorporate embedded fillers into thermoregulatory textiles; and (3) the utilization of knitting or weaving technologies to create thermal-regulating textiles. Recent advancements have led to the development of specialized textiles aimed at enhancing heat dissipation in the human body through various scenarios (e.g., daytime-, evaporative-, and responsive-radiative cooling), as demonstrated in Fig. 8. The invention of thermal-regulating textiles of ERCS is emergent, aimed to provide optimal thermal comfort in response to increasing demands [167–169].

### Daytime-Radiative Cooling Textiles

The creation of cost-effective and energy-efficient radiative cooling clothing suitable for practical application continues to pose a considerable challenge in various scenarios, due to the prevalence of radiative cooling materials that tend to absorb incident solar radiation [170, 171]. Consequently, it is essential to develop high-efficiency cooling textiles that exhibit enhanced solar reflectivity in the solar spectrum to restrict solar radiation while maintaining excellent MIR radiation to enhance thermal radiation dissipation [172, 173]. For example, some inorganic or organic compounds (e.g., ZnO and Al_2_O_3_) have been utilized to enhance solar reflectance. Moreover, the DTRC effect is realized via integrating metamaterials or hierarchical structures, which facilitate the design and fabrication of textiles that prevent solar energy input while effectively emitting in the MIR range [174–177].

**Fig. 9:**
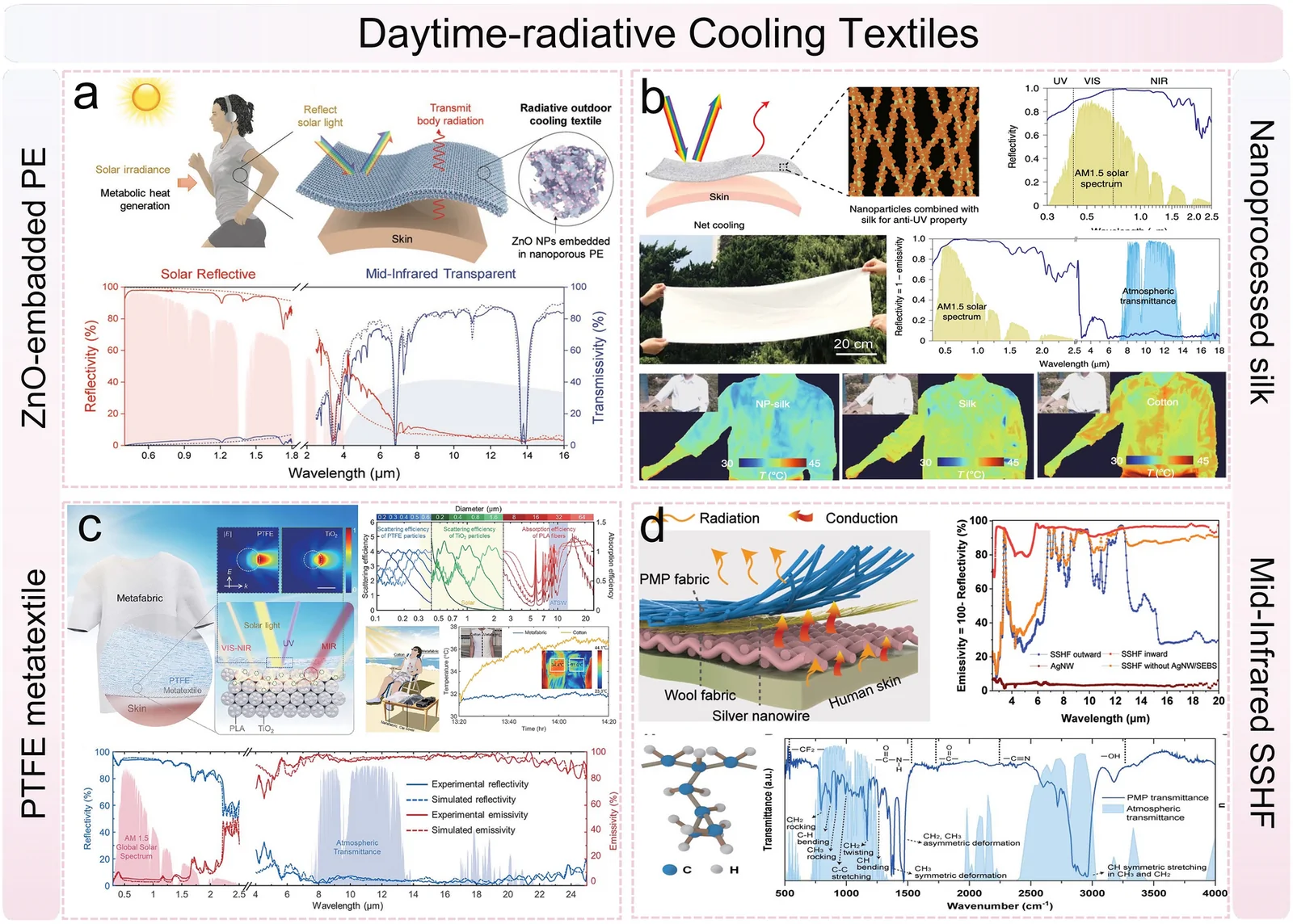
Daytime-radiative cooling textiles. **a** Schematic of the ZnO nanoparticle-embedded nanoporous PE textile, and optical spectra of ZnO-PE with the range of 0.3–16 µm. Reproduced with permission [174]. Copyright 2018, Wiley–VCH. **b** Schematic of subambient daytime radiative cooling design for nanoprocessed silk, and the reflectivity spectrum of nanoprocessed silk in the 0.3–18 μm wavelength range. Reproduced with permission [175]. Copyright 2021, Springer Nature. **c** Schematic of a metafabric for daytime radiative cooling, and measured optical spectra of the metafabric (0.3 µm to 25 µm). Reproduced with permission [176]. Copyright 2021, The American Association for the Advancement of Science. **d** Spectrally engineered textile for radiative cooling against urban heat islands. Reproduced with permission [177]. Copyright 2024, The American Association for the Advancement of Science

A method that efficiently and economically provides localized outdoor cooling for the human body in a zero-energy input manner has been demonstrated. Cai et al. [174] introduced a novel spectrally selective nanocomposite textile for radiative outdoor cooling, utilizing zinc oxide nanoparticle-embedded polyethylene (ZnO-embedded PE), as exhibited in Fig. 9a. This ZnO-embedded PE reflects over 90% of solar irradiance while selectively transmitting thermal radiation for human body, rendering a temperature reduction of 5–13 °C in comparison with conventional textiles. This garment functions as a radiative cooler, exhibiting exceptional passive cooling effects and suitability for large-scale production, indicating considerable potentials to societal sustainability in both health and economic sectors [174]. To inhibit the intrinsic absorption of protein in the ultraviolet spectrum of natural silk, another inorganic alloyed Al_2_O_3_ particle was demonstrated, which incorporated into a natural silk material to effectively achieve the DTRC effect. Zhu et al. [175] developed a nanoprocessed silk utilizing a molecular bonding design and a scalable coupling reagent-assisted dip-coating technique, achieving a remarkable DTRC effect with a temperature reduction of 8 °C compared to natural silks (Fig. 9b). The enhanced cooling effect was ascribed to the high-refractive-index capability of Al_2_O_3_ to obstruct ultraviolet light and the inherent high emissivity of silk in the MIR wavelength range. This approach of customizing natural fabrics using scalable nanoprocessed techniques gives new insights into developing thermoregulatory textiles [175].

To balance the passive cooling effect and scalable practicality, Zeng et al. [176] developed large-scale woven meta-fabrics composed of a titanium oxide–polylactic acid (TiO_2_-PLA) composite textile, bonded with a thin layer of polytetrafluoroethylene (PTFE). The PTFE meta-textile exhibited a high emissivity of 94.5% in the MIR range and a high reflectivity of 92.4% in the solar spectrum, as illustrated in Fig. 9c. A person enveloped in this PTFE meta-textile could achieve a lower temperature of 4.8 °C than that conventional cotton fabrics counterpart. This result was ascribed to the hierarchical morphology of the randomly distributed scatterers inside the PTFE meta-textile. Despite the potential to radiative cooling clothing for alleviating personal thermal discomfort amid rising global temperature, urban locations exhibited heat island effects that considerably diminished the efficacy of cooling textiles, due to the absorption of emitted radiation from the ground and adjacent buildings [176]. To investigate this issue, Wu et al. [177] presented a MIR spectrally selective hierarchical fabric (SSHF) via molecular design that exhibited greatly boosted emissivity in the thermal radiation region of human body, thus reducing net heat absorption from the ambient environment (Fig. 9d). This engineered SSHF was integrated PMP fabric, AgNWs, and a wool fabric layer into a multilayered construction, exhibiting a high reflectivity of 0.97 in the solar spectrum, ascribed to strong Mie scattering from the nano-micro hybrid fibrous structure. Furthermore, this selective-spectrum design for vertically oriented textiles presents an innovative and efficient solution to mitigate the urban heat island effect, potentially reducing energy usage for air conditioning and performing as a precautionary strategy against heat-induced health issues [177].

### Evaporative-Radiative Cooling Textiles

The development of functional fabrics that provide cooling performance is crucial for maintaining human thermal comfort during their daily events. Although these thermoregulatory textiles offer effective cooling performance, they still suffer various thermal constraints in practical applications [128, 178, 179]. Excessive sweat can lead to garments adhering to the skin surface of human body, resulting in chills that undermines thermal comfort. It is imperative to create fabrics that improve thermal comfort and productivity while facilitating effective sweat evaporation and heat dissipation [180, 181]. To develop the sweat-inducing and thermoregulation-efficient textiles for practical uses, particular constructions have been designed to enhance sweat transport from the human body, including optimized PE structures and sandwich-structured textiles, both while demonstrating effective cooling effects [182, 183].

**Fig. 10:**
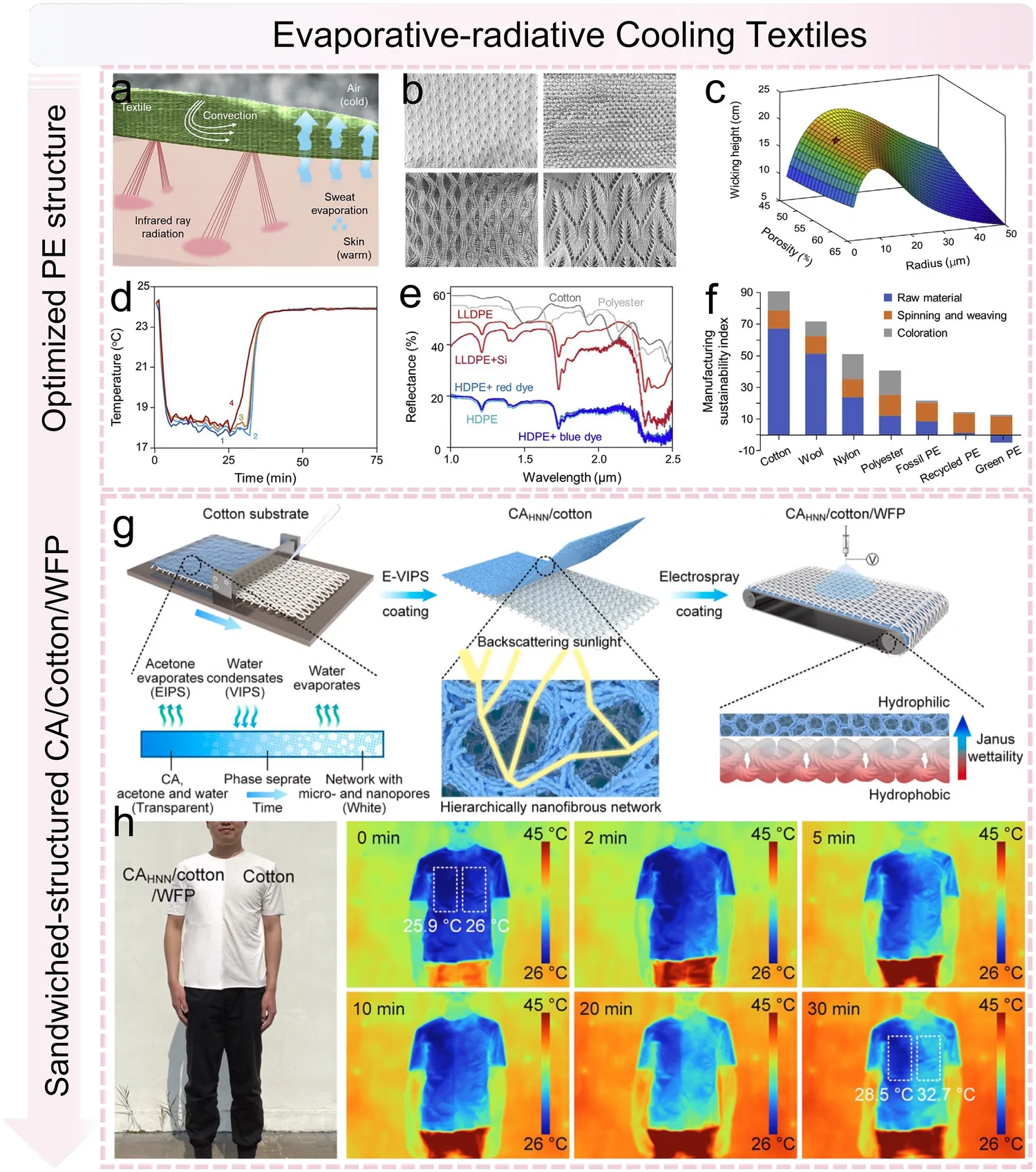
Evaporative-radiative cooling textiles. **a** Depiction of thermal energy and mass transmission. **b** Diverse designs of woven PE fabrics. **c** Structural optimization of PE fabrics for enhanced wicking performance. **d** Temperature records for various time. **e** Reflectivity of various textiles in the MIR range. **f** Comparisons of sustainable index of different textiles. Reproduced with permission [182]. Copyright 2021, Elsevier. **g** Schematic representation for the preparation of sandwich-structured CA/cotton/WFP textiles including a chemically nanofibrous network and Janus wettability. **h** Optical and infrared camera images of a volunteer for various textiles. Reproduced with permission [183]. Copyright 2022, Elsevier

To fully maximize energy savings through efficient wicking and drying, Fang et al. [182] engineered smart PE textiles through structural optimization to enhance their evaporative and radiative cooling capabilities, as illustrated in Fig. 10a. The remarkable flexibility of PE fibers was demonstrated, indicating its adaptability for being tailored to various constructions in Fig. 10b. The optimization of single-material PE textile structures could facilitate the process of evaporative cooling, leading to efficient wicking performance (Fig. 10c). Also, the fully optimized PE textile demonstrated enhanced overall cooling effects (radiative and evaporative models), achieving a temperature reduction of 5 °C, attributed to its quick water propagation and concurrent rapid evaporation, as exhibited in Fig. 10d [182]. Moreover, the near-infrared spectral fingerprint of PE textiles was unaffected by colorants (e.g., dyes and embedded nanoparticles), thus facilitating automatic recycling, as shown in Fig. 10e. Simultaneously, the sustainability index of PE textiles was thoroughly assessed during the production phase, usage phase, and end-of-life phase (Fig. 10f). These findings may offer a scientific direction for the advancement of alternative sustainable smart fabrics [182].

Another structured textile featuring a hierarchically nanofibrous network and Janus wettability for evaporative-radiative cooling has been introduced by Miao and co-workers [183]. It was aimed at reducing the buildup of heat and preventing excessive perspiration to the skin surface of human body. This sandwich-structured CAHNN/cotton/WFP textile was synthesized via a combined phase separation process, followed by electro-spraying the WFP emulsion on the pre-treated textile that contained hydrophobic perfluoroalkyl segments (Fig. 10g). This sandwich-structured CAHNN/cotton/WFP textile exhibited exceptional optical properties, showcasing a notable solar reflectivity of 93.4% and a striking MIR emissivity of 96.3% [183]. Furthermore, it demonstrated a remarkable one-way transport index of 1140%, indicating an outstanding capacity for directional water transport. In a practical circumstance, a human body enveloped by this sandwich-structured textile achieved a temperature decrease of ~ 4.2 °C in comparison with a conventional cotton counterpart, as shown in Fig. 10h. Thus, this design facilitated a substantial decrease in human perspiration and excessive thermal stress in humid conditions. And the effective production of these evaporative-radiative cooling textiles established a conducive microclimate for the human body, addressing the increasing needs for improved efficiency and sustainability [183].

### Responsive-Radiative Cooling Textiles

Clothing, regarded as the “second skin” of the human body, is indispensable in daily life and plays a crucial role in maintaining thermal comfort by regulating heat dissipation and insulation. Recently, the ERCSs offer an energy-efficient cooling approach by effectively dissipating substantial heat from the human skin to the external environment via thermal radiation, while minimizing solar energy absorption, thereby enabling temperature decrease below its environments. However, implementing responsive systems to regulate radiative cooling effect in textiles presents significant challenges [184, 185]. Such as the study reported by Lan et al. [186], it demonstrated the potential of thermoregulatory textiles to combine photothermal conversion and humidity-responsive abilities, achieving effective thermal comfort in dynamic environments. Although certain engineered designs, such as photonic crystals and composite materials, have effectively achieved radiative cooling performance, these designs are insensitive to environmental fluctuations and lack a proficient prerequisite for responsive regulation of radiative cooling effects [18, 181, 185]. As a result, substantial advancements have been achieved in the creation of effectively responsive radiative cooling performance, including infrared-gated optical channels and thermal-triggered transmission channels, expanding their practical uses in various scenarios [187, 188].

**Fig. 11:**
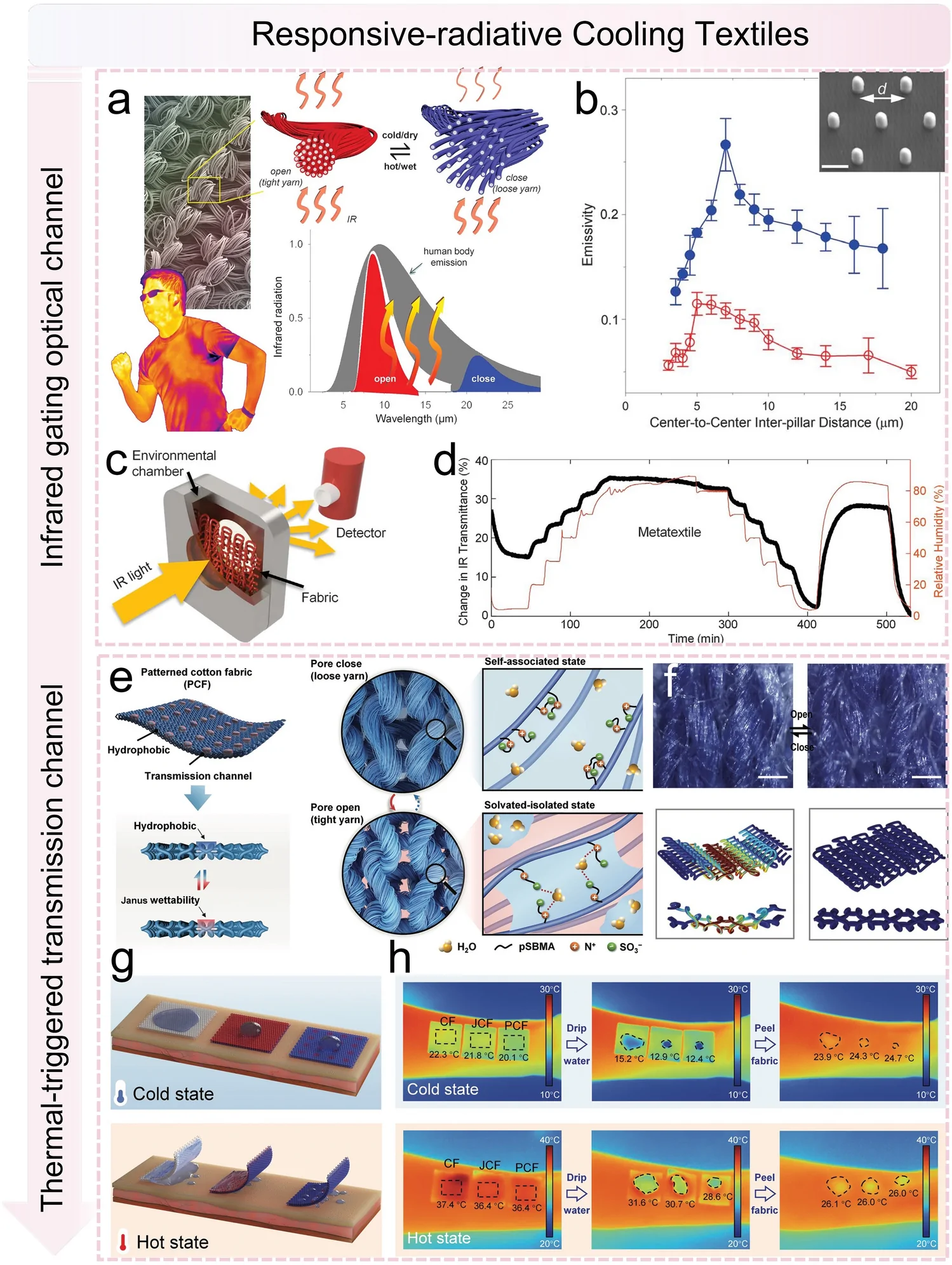
Responsive-radiative cooling textiles. **a** Design concepts of an infrared gating textile. **b** Distance dependence of the emissivity of carbon (blue) and gold-coated (red) pillar arrays. Inset: SEM image of a localized region of a pillar array with a scale bar of 5 µm. **c** Schematic setup for experimental measurements. **d** Infrared gating of the metatextile. Reproduced with permission [187]. Copyright 2019, The American Association for the Advancement Science. **e** Schematic illustration of the PCF with thermal-triggered transmission channels. **f** Optical microscope images and structure simulation in two states. **g** Schematic illustration and **h** infrared camera images of textiles in two states. Reproduced with permission [188]. Copyright 2023, Wiley–VCH

To possess the ability to dynamically regulate the optical channel for thermoregulations, Zhang et al. [187] created an infrared-responsive textile via implementing triacetate-cellulose bimorph fibers with a thin layer of conductive materials (e.g., carbon nanotubes). This infrared-gated textile effectively managed heat radiation for responsive thermoregulations, grounded in several fundamental principles, as illustrated in Fig. 11a. Each textile yarn was composed of a collection of microfibers functioning as meta-component and a responsive mechanism, adept at reacting to diverse skin surroundings [187]. Furthermore, the overall emissivity of each carbon column array exhibited variation with respect to column positioning, as established through computational analysis. This highlighted the notable nonlinear optical coupling effect among the carbon columns within the array (Fig. 11b). A systematic experiment was carried out to characterize their thermal infrared response at regulated humidity levels (Fig. 11c). It was worth noting that the infrared-gated textile exhibited a relative shift in MIR transmittance of up to 35.4% (Fig. 11d). This outcome facilitated the creation of autonomous and localized thermoregulatory technologies, while strengthened our ability to acclimatize to challenging scenarios [187].

A further strategy focused on the responsive thermoregulation of moisture in textiles was developed, which was sought to address the issues faced by existing pore-actuated fabrics, encountering significant macro-dimensional deformation and lacking the ability to regulate the trajectory and rate of sweat transfer. Lin et al. [188] introduced a patterned cotton fabric (PCF) by developing thermal-triggered transmission channels on knitted hydrophobic cotton, capable of autonomously altering channels in response to ambient temperature (Fig. 11e). In cold conditions, the channels became “closed” ascribed to the self-association of SBMA chains. This transmission rendered hydrophobic channels and fluffy yarn, which obstructed rainfall input and diminished water vapor exchange. At elevated temperatures, the dipolar connections were interrupted, resulting in solitary polymer chains to become fully solvated, facilitating the rapid release of water vapor and the unidirectional transport of sweat from the triggered channels (Fig. 11f) [188]. To further illustrate the thermoregulatory effects of this PCF, two conditions were examined: hot and cold scenarios. In cold state, the same quantity of water was placed onto the outer surface of fabrics. When hot, the textiles facilitated the expulsion of water droplets like the process of sweating (Fig. 11g). In contrast to unblemished cotton, the PCF featuring thermal-triggered transmission channels possessed the capability to modify its wettability in response to surrounding temperatures, effectively preventing the intrusion of external water droplets and enhancing heat retention with a higher temperature of 0.8 °C in cold conditions, while facilitating directional sweat transport at high temperatures [188] (Fig. 11h). This outcome demonstrated that the PCF with waterproof capability at low temperatures and unidirectional water permeability at high temperatures might effectively modulate its moisture levels to the human skin, hence optimizing skin thermal comfort in various circumstances.

Thermal-regulating textiles are constructed from polymers (e.g., nylon, polyester) and specialized fibers designed for elevated MIR emissivity and adaptive solar reflectance [165]. Thermoregulatory fabrics incorporate reflective chemicals (e.g., TiO₂, SiO₂ nanoparticles) to improve sun reflectivity while preserving comfort in wear. The emissivity of MIR is adjusted via material selection, such as the humidity-dependent moderate emissivity of acrylic, and surface changes, like nanoporous polyethylene layers that facilitate the transmission of body heat [188]. Responsive fabrics enhance emissivity across several environments, including moisture management [184]. These fabrics exhibit durability, breathability, and washability, rendering them suitable for personal thermal regulation.

## Energy-Saving Devices of ERCSs

**Fig. 12:**
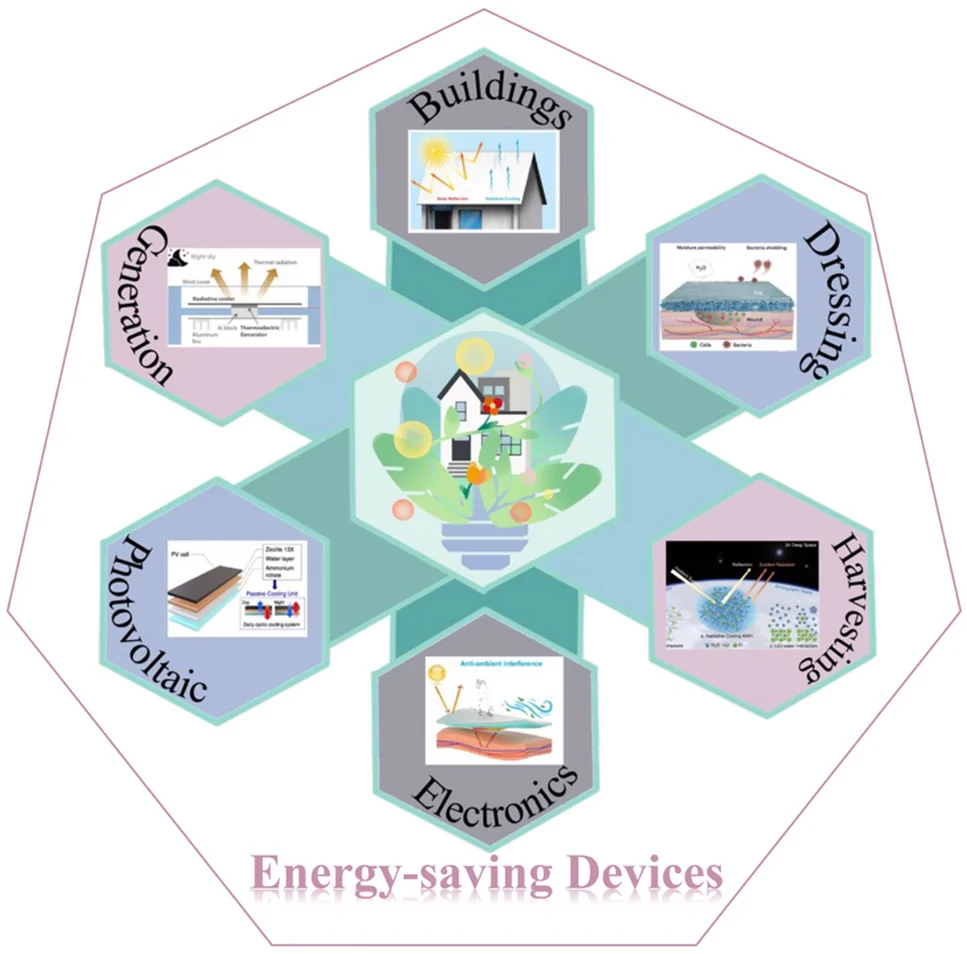
Schematic of energy-saving devices, including buildings, dressing, harvesting, electronics, photovoltaic, and generation. Reproduced with permission [200]. Copyright 2019, The American Association for the Advancement of Science. Reproduced with permission [201]. Copyright 2024, Springer Nature. Reproduced with permission [202]. Copyright 2024, Springer Nature. Reproduced under terms of CC-BY 4.0 license [203]. Copyright 2023, The American Association for the Advancement of Science. Reproduced under the terms of CC-BY license [204]. Copyright 2023, Springer Nature. Reproduced with permission [205]. Copyright 2019, Elsevier

Based on the previously discussed principles of engineered radiative cooling devices, it is acknowledged that an effective radiative cooling effect requires a radiator to exhibit both high solar reflectance to minimize solar absorption and significant thermal MIR emittance within the ATW to effectively dissipate excess heat to outer space [189–192]. Radiative cooling has significant efficacy for energy-saving technologies, rendering it appropriate for various practical applications [193–199]. This section summarizes the contemporary applications of recently described radiative coolers, including buildings, wound dressing, water harvesting, electronics, photovoltaics, and generation for energy-saving devices, as shown in Fig. 12 [200–205].

**Fig. 13:**
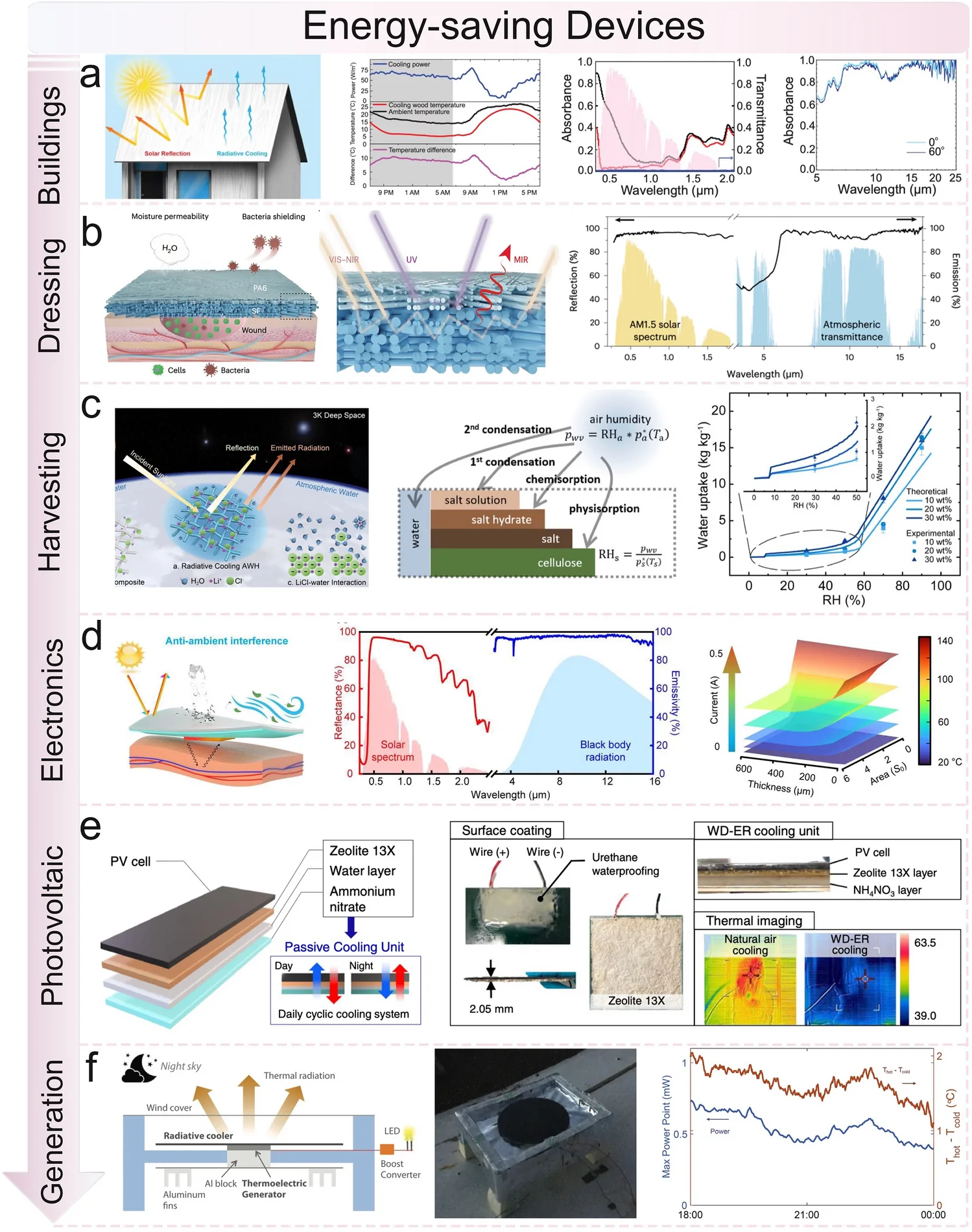
Energy-saving devices of ERCS **a** A radiative cooling structural material of building. Reproduced with permission [200]. Copyright 2019, The American Association for the Advancement of Science. **b** Radiative cooling dressings for wound healing. Reproduced with permission [201]. Copyright 2024, Springer Nature. **c** Radiative cooling sorbent for water harvesting. Reproduced under the terms of CC-BY license [202]. Copyright 2024, Springer Nature. **d** Ultrathin, soft, radiative cooling interfaces in skin electronics. Reproduced under the terms of CC-BY 4.0 license [203]. Copyright 2023, The American Association for the Advancement of Science. **e** Self-recovering passive cooling for photovoltaic cell. Reproduced with permission [204]. Copyright 2023, Springer Nature. **f** Generating Light from Darkness. Reproduced with permission [205]. Copyright 2019, Elsevier

To mitigate human dependence on energy-inefficient cooling techniques, particularly HVAC systems in edifices, would significantly pose a threat to energy issues [199, 206–209]. Li et al. [200] produced a multifunctional, passive radiative cooling structural material through total delignification and densification of wood, showcasing reinforced mechanical performance with a remarkable strength of 404.3 MPa. The radiative cooling wood demonstrated enhanced whiteness, mainly attributed to the minimal optical loss of the cellulose fibers and its disorganized photonic architecture. Their designed cellulose nanofibers were endowed with high reflectivity in the solar spectrum and high emissivity in MIR wavelengths, leading to sustained subambient cooling effect both at night and during the day, with average below-ambient temperatures exceeding 9 and 4 °C, respectively. This result rendered an average cooling power of 53 W m^−2^ during a 24-h period. This scalable approach for versatile, cooling-wood material facilitates future energy-efficient and practical sustainability toward thermoregulatory applications, significantly decreasing greenhouse gas emissions and energy usage [200] (Fig. 13a).

The creation of sophisticated wound dressings with thermal regulation is essential, as these wound dressings are vulnerable to local environmental disturbances and oxidative stress, potentially resulting in increased wound temperatures when exposed to sunlight [210, 211]. Zhu et al. [201] proposed a daytime-radiative cooling dressing with a bilayer fiber composed of polyamide 6 and silk fiber (PA_6_/SF), which could modify the local wound microenvironment for expediting wound healing in outdoor. The engineered PA6/SF nanofibers exhibited an improved MIR emissivity of 0.94 and a significant solar reflectivity of 0.96, leading to a decreased temperature of ~ 7 °C compared to the ambient temperature under the condition of sunlight. Furthermore, the PA_6_/SF nanofibers, implemented in the repair of full-thickness mouse skin injuries in sunlight, achieved a more rapid process of wound healing than those conventional dressings counterpart. This outcome could be attributed to the reduction of oxidative stress within the wounds, which consequently led to a restriction of inflammation. Therefore, this innovative research presents a valuable approach for passive thermoregulation aimed at facilitating wound healing in sunlight conditions [201] (Fig. 13b).

Widely recognized atmospheric water harvesting systems (e.g., condensation or adsorption-based) predominantly depend on a singular mechanism constrained by operational conditions and suboptimal performance [212–214]. In the pursuit of a straightforward and scalable fabrication process that delivered outstanding performance, Zhu et al. [202] combined various mechanisms, like thermal adsorption effects, radiative cooling performance, and multiscale cellulose–water interactions, to efficiently enhance water harvesting effects across a relative humidity range of 8% to 100%. Moreover, the collected water promoted radiative cooling properties due to its inherently high emissivity, leading to improved sorption and condensation with low energy expenditure. Theoretical models, which integrated multiple adsorption processes and considered the impacts of radiative cooling, had been validated, providing accurate predictions of actual water uptake and clarifying the interplay between composite–water–energy relationships. The collaborative activities produce a remarkably promising and scalable strategy for an atmospheric water harvesting system that possesses a high production rate, cost-effectiveness, and environmental sustainability, boosting to develop a compelling solution to the issue of water shortage, especially in dry regions [202] (Fig. 13c).

Thus far, the regulation of temperature has been crucial in the field of electronics, particularly concerning the development of wearable and skin electronics, since it definitely influences the degree of integration, adaptability, and compactness of these electronic devices [215, 216]. Designing and integrating a material that exhibiting effectively radiative cooling effect while also facilitating adequate non-radiative heat transfer, particularly in a straightforward approach, could represent a groundbreaking advancement in the thermal management of skin electronics [217, 218]. Consequently, Li et al. [203] described a thermoregulatory approach for skin electronics, employing an ultrathin, flexible radiation-cooled interface that effectively enhanced the cooling effect in electronic devices through both radiative and non-radiative heat transfer, resulting in a temperature reduction exceeding 56 °C compared to the unprotected electronic device at 90 °C. This finding presents a promising potential for enhancing thermoregulatory performance in sophisticated skin interface electronics for multifunctional and wireless healthcare monitoring applications [203] (Fig. 13d).

Drawing from current studies on passive cooling techniques toward photovoltaic cells [219–223], Kim et al. [204] created a self-recovery passive cooling device constructed from cost-effective materials, with forested both power generation efficiency and longevity of the cells. Water-saturated zeolite 13X was applied to the rear of the solar cell. Upon the application of heat, water was desorbed from zeolite 13X (latent cooling) and started to dissolve ammonium nitrate, thus initiating a heat absorption process for cooling purposes. Moreover, this was a reversible procedure that could self-recover at nighttime. The device operated on the concept that the water-absorbing characteristics of porous materials were inversely related to temperature. The average temperature of the solar cells decreased by 15.1 °C, the cooling energy density attained 2876 kJ kg^−1^, and the average cooling power was 403 W m^−^2. These results demonstrated that effective passive cooling effects could be achieved with cost-effective materials for solar cells [204] (Fig. 13e).

Consistent access to energy continues to pose a significant challenge, particularly in off-grid regions globally [224, 225]. Although solar cells facilitated distributed power generation throughout the day, there is currently no comparable option available during the night, whereas the capacity to produce energy during nighttime hours can significantly facilitate various practical applications (e.g., illumination and low-energy sensors) [226]. Thus, Raman et al. [205] revealed a cost-effective, modular system allowed for producing substantial quantities of electricity during nighttime, harnessing the cold of space via radiative cooling techniques. This innovative electrical generation, in contrast to traditional thermoelectric generators, connects the cold side of a thermoelectric module to a surface oriented toward the sky, allowing it to radiate heat into the frigid expanse of space. Meanwhile, the warmer side absorbed heat from the ambient surroundings, rendering electricity production during nighttime. As shown from conducted experiments, it showcased power generation of 25 mW m^−^2 by lighting generator and affirmed a model that accurately reflected the efficiency of the device [205] (Fig. 13f).

Energy-saving devices that employ ERCSs are implemented in numerous sectors, such as buildings, electronics, electricity generation, and water harvesting, leading to a notable decrease in energy consumption. Structures and electronic devices utilizing ERCS technology, including radiative cooling coatings and window films, effectively diminish cooling demands by reflecting sunlight and emitting thermal radiation [15]. Thermoelectric generators that incorporate radiative coolers effectively capture energy from variations in temperature. Radiative cooling facilitates the process of atmospheric water harvesting through the condensation of moisture on surfaces that are cooler than the surrounding environment. Maximizing solar reflectivity involves the use of metamaterials such as Ag/SiO₂ layers, along with photonic bandgap engineering and photoluminescent carbon dots. Meanwhile, enhancing MIR emissivity can be achieved through materials exhibiting vibrational absorption, such as C–O–C bonds in cellulose acetate, as well as through textured or porous surfaces like hierarchically structured ceramics and 2D gratings or meta-structured coatings, including silica aerogels [138, 215].

Furthermore, ERCSs are sophisticated technologies that achieve passive cooling effects of objects through the emission of thermal radiation to outer space within the atmospheric transparency window (8–13 µm), while also reflecting solar radiation (0.3–2.5 µm). The dual optical characteristics of ERCSs facilitate temperature reduction without the need for external energy input, offering an environmentally sustainable solution for thermal-regulating and energy-saving applications, with their effectiveness dependent on materials designed for specific requirements [13, 48]. For the human-related realm of textiles and wearables, key priorities encompass thermal comfort and functionality, requiring the use of flexible, breathable, and waterproof materials that exhibit high solar reflectance and robust infrared emissivity for effective radiative cooling effects [38, 176]. Buildings’ development prioritizes scalability and durability, necessitating materials with superior solar reflectance, significant infrared emittance, self-cleaning properties, and esthetic flexibility, including various color options [207, 208]. Additional applications include electronics cooling, necessitating compatibility of ERCSs with superior cooling properties; power generation, which requires a balance between emissivity and insulation for effective energy harvesting; and preservation systems, which require wear-resistant materials for the efficient cooling of items such as food or ice [47, 215, 219, 221]. Future advancements rely on the optimization of application-specific requirements, alongside improvements in performance, durability, and cost-effectiveness, to facilitate broader adoption of ERCSs and support global sustainability efforts.

## Summary and Outlook

Thermoregulation has increasingly been crucial as society has evolved throughout time. ERCSs get considerable focus due to their importance in efficient thermoregulation. Herein, a multitude of developments in creative technologies for radiative cooling effects have been analyzed, including biomimetic designs, chromatic materials, metastructured constructions, and multilayered architectures. Additionally, we explored the burgeoning applications of ERCSs in thermo-regulating fabrics and energy-saving devices. Thermoregulatory textiles, primarily concerning several functions (daytime-, evaporative-, and responsive-radiative cooling), were developed to enhance thermal comfort for the human body in various circumstances. Moreover, energy-efficient devices across many applications (e.g., building, wound dressing, water harvesting, electronics, photovoltaics, and power generation) were delineated, highlighting their remarkable cooling capabilities and their role in alleviating the energy crisis and reducing carbon emissions. ERCS’ techniques and applications have experienced significant improvements in recent years due to these remarkable breakthroughs. Lastly, the remaining major challenges and associated research direction were presented to advance the practical applications of ERCSs.

The rapid advancement in ERCS has transitioned the emphasis from merely boosting optical effects to tackling practical application difficulties. These issues encompass the maximized thermoregulatory effect, environmental adaptability, scalability and sustainability, as well as interdisciplinary integration. Notwithstanding considerable endeavors, a complete evaluation summarizing the numerous methodologies of ERCS and evaluating their efficacy and applicability remains absent. This review seeks to close this gap by methodically summarizing recent advancements in the practical applications of ERCSs and motivates scholars and practitioners to innovate technologies that are both theoretically rigorous and commercially viable.

In light of the development in radiative cooling technologies, several challenges remain to be addressed, along with opportunities to explore in the subsequent four areas [227–229]:

1. Maximized Thermoregulatory Effect. The effectiveness of radiative cooling is fundamentally dependent on the precision of optical performance achieved through radiation-controlled mechanisms, which are designed to minimize solar heat absorption and maximize the dissipation of MIR thermal radiation. Strategies to enhance radiative cooling effects encompass: (1) incorporating nanoparticles into the cooler to achieve improved reflectivity in the solar spectrum, thereby reducing heat absorption; (2) refining cooler designs, including meta-structures and nano-macro configurations, to enhance emissivity in the MIR range, thus facilitating infrared radiation dissipation; and (3) integrating additional heat dissipation mechanisms within coolers, such as conduction- and evaporation-controlled. Optimally, the maximized cooling effects of ERCS in a zero-energy-consumption method can be designed through integrating total strategies before-mentioned for practical applications [13, 19, 230].
2. Environmental Adaptability. The development of radiative cooling systems capable of accurately adapting to environmental conditions (e.g., temperature, humidity) can enhance their functionality beyond a singular cooling application, facilitating all-weather performance. To date, the designed materials and systems exhibiting responsive optical characteristics are constrained. Consequently, it is essential to investigate novel materials and techniques to develop responsive coolers that allows for adapting to the changes of environmental circumstances [184, 185, 231].
3. Scalability and Sustainability. RCSs are currently being utilized in numerous applications in outdoor scenarios, such as buildings, vehicles, and textiles. Cost-effectiveness and scalable production are critical determinants in assessing the potential for commercializing emerging technologies of ERCSs. Moreover, contemporary systems exhibit inadequate sustainability of fabrication process concerning green materials, effective procedures, and carbon emission, all of which are essential for practical applications. Joint collaboration among academia, industry, and research organizations is crucial for converting radiative cooling technology research into practical and sustainable applications [232, 233].
4. Interdisciplinary integration. Despite extensive utilization in buildings, vehicles, and textiles, the integration of ERCSs into various interdisciplinary applications, including batteries, sensors, and healthcare, poses distinct challenges. Moreover, AI-assisted devices would be integrated with advanced RCSs and linked to analytical data processing on mobile phones through Bluetooth. This integration aims to create next-generation for temperature-regulating textiles and energy-saving devices that can execute a range of functions for the real-world applications, such as detection and analysis. It is our aspiration that this review will inspire additional groundbreaking research on ERCS and encourage its advancement across diverse fields [211, 218, 234, 235].
5. Overall, the advancements in ERCS have been significantly achieved through the innovative design of materials and structures, enhancing thermoregulatory effects and expanding their scope of applications, while challenges and opportunities are present in this evolving domain. Through the enhancement of technical innovations, the refinement of commercial procedures, and the assurance of efficient integration, we may render ERCS as a feasible alternative for future thermoregulatory solutions, both effectively and practically.
